# Biomonitoring Study of Toxic Metal(loid)s: Levels in Lung Adenocarcinoma Patients

**DOI:** 10.3390/toxics12070490

**Published:** 2024-07-04

**Authors:** Nataša Milošević, Maja Milanović, Danica Sazdanić Velikić, Jan Sudji, Jelena Jovičić-Bata, Milorad Španović, Mirjana Ševo, Mirka Lukić Šarkanović, Ljilja Torović, Sanja Bijelović, Nataša Milić

**Affiliations:** 1 Department of Pharmacy, Faculty of Medicine, University of Novi Sad, Hajduk Veljkova 3, 21000 Novi Sad, Serbia; natasa.milosevic@mf.uns.ac.rs (N.M.); jelena.jovicic-bata@mf.uns.ac.rs (J.J.-B.); ljilja.torovic@mf.uns.ac.rs (L.T.); natasa.milic@mf.uns.ac.rs (N.M.); 2 Institute for Pulmonary Diseases of Vojvodina, Clinic for Pulmonary Oncology, Faculty of Medicine, University of Novi Sad, 21204 Sremska Kamenica, Serbia; danica.sazdanic-velikic@mf.uns.ac.rs; 3 Institute of Occupational Health Novi Sad, Faculty of Medicine, University of Novi Sad, 21000 Novi Sad, Serbia; jansudy@yahoo.com (J.S.); milorad.spanovic@mf.uns.ac.rs (M.Š.); 4 Faculty of Medicine, University of Novi Sad, 21000 Novi Sad, Serbia; 902012d23@mf.uns.ac.rs; 5 IMC Banja Luka-Center of Radiotherapy, Part of Affidea Group, 78000 Banja Luka, Bosnia and Herzegovina; 6 Clinical Center of Vojvodina, Clinic for Anesthesiology, Intensive Therapy and Pain Therapy, Faculty of Medicine, University of Novi Sad, 21000 Novi Sad, Serbia; mirka.lukic-sarkanovic@mf.uns.ac.rs; 7 Institute of Public Health of Vojvodina, Faculty of Medicine, University of Novi Sad, 21000 Novi Sad, Serbia; sanja.bijelovic@mf.uns.ac.rs

**Keywords:** arsenic, cadmium, chromium, mercury, nickel, lung cancer

## Abstract

Lung cancer is a leading cause of cancer deaths worldwide. The aim of this study was to investigate heavy metal(loid)s (Cd, Pb, Hg, Cr, Mn, Mo, Ni, and As) in lung cancer patients in order to elucidate their role as lung cancer environmental risk factors. Sixty-three patients of both sexes with adenocarcinoma stage IIIB or IV were enrolled in this research. The heavy metal(loid) urine concentrations were measured using ICP-MS. Arsenic was quantified above 10 μg/L in 44.44% of the samples. Nickel urinary concentrations above the ToxGuide reference levels were found in 50.79% of the samples, while lead was quantified in 9.52% of the urine samples. The urinary chromium levels were above the mean ToxGuide levels in 41.27% of the patients and were significantly higher in men in comparison with women (*p* = 0.035). The chromium urinary concentrations were positively associated with the CRP serum levels (*p* = 0.037). Cadmium was quantified in 61.90% of the samples with levels significantly higher in females than in males (*p* = 0.023), which was associated with smoking habits. Mercury was measured above the limit of quantification in 63.49% of the samples and was not associated with amalgam dental fillings. However, the Hg urinary concentrations were correlated positively with the ALT (*p* = 0.02), AST (*p* < 0.001), and GGT (*p* < 0.001) serum levels. In 46.03% of the samples, the Mo concentrations were above 32 μg/L, the mean value for healthy adults according to the ToxGuide, and 9.52% of the patients had Mn levels higher than 8 μg/L, the reference value for healthy adults based on ToxGuide data. The obtained results are preliminary, and further studies are needed to have a deeper insight into metal(loid) exposure’s association with lung cancer development, progression, and survival prediction.

## 1. Introduction

Lung cancer is a leading cause of all cancer-related deaths (18.0% of the total cancer deaths) in both sexes and the second most common cancer in men [1]. The socioeconomic burden of lung cancer morbidity and mortality affects global healthcare systems. The rapidly growing incidence of lung cancer is a result of aging, but it also reflects the distribution of the main risk factors for cancer development, such as tobacco smoking and air pollution. Hungary had the highest overall rate of both lung cancer incidence and mortality in 2020 followed by Serbia, while Turkey had the highest overall rate of both lung cancer incidence and mortality in 2020 in men, again followed by Serbia [2]. Adenocarcinoma is the most common subtype of non-small lung cancers and accounts for approximately 40% of lung cancers [3]. Lung adenocarcinoma evolves from the glandular cells which produce mucus and support the structural lung integrity. Tobacco smoking is the main risk factor for the initiation and progression of all non-small histological types of lung cancer, including adenocarcinoma [4]. Patients with smoking histories are at a 20-fold higher risk for lung cancer in comparison with people who never smoked, with the duration of continuous smoking as one of the most important determinants for lung cancer development [5]. Genetic predisposition, occupational exposure to asbestos, silica, and radon, as well as air pollution and poor diet are recognized as risk factors for lung cancer which act independently or mutually with tobacco smoking [6]. Surprisingly, the global incidence of lung cancers among people who never smoked has remained unchanged or even increased [7], emphasizing the need for epidemiological studies in this area.

Metal(loid)s are naturally occurring elements which can be found in the atmosphere, Earth’s crust, and water bodies. Anthropogenic activities, such as fossil fuel combustion, mining and smelting activity, metal processing in refineries, industrial processes, wood preservation, paper processing, and domestic and agricultural use of metals and their compounds, have distributed metal-containing compounds throughout the environment. Metal corrosion, soil erosion, the resuspension of sediments, atmospheric deposition and evaporation from water, leaching of heavy metals from sediments into ground waters, and volcanic eruptions are also attributed to the heavy metals and metalloids cycling in the environment [8]. Heavy metals and metalloids are transferred from the abiotic environment (soil, water, and air) in living organisms, where they accumulate and enter the food chain [9,10]. Once metal(loid)s enter the human body though ingestion, inhalation, or dermal absorption, they are bonded to proteins or nucleic acids and accumulate in body cells and tissues. Depending on the dose, route, and time of exposure, metal(loid)s cause a spectrum of harmful effects on physiological processes. They can damage the protein and nucleic acid structure, derange the cellular function, and mimic the hormone affects, thus causing disruption of the endocrine, immune, and reproductive systems and finally enhancing cancer development and progression [11].

The exponential increase in industrial, agricultural, and technological development has led to continuous chronic environmental and occupational exposure to heavy metals such as lead (Pb), cadmium (Cd), chromium (Cr), nickel (Ni), manganese (Mn), molybdenum (Mo), and mercury (Hg) and heavy metalloids such as arsenic (As) [12,13,14,15]. There is a substantial global health care concern regarding adverse health effects, especially the carcinogenic potential of chronic heavy metal exposure [15]. The Agency for Toxic Substances and Disease Registry (ATSDR) recognizes substances which present the most significant potential threat to human health and rank them according to their frequency, toxicity, and potential human exposure, combined with the National Priorities List (NPL). The NPL ranks arsenic first on the list, with lead being in second, mercury third, and cadmium in seventh [16]. Although not listed on the NPL, heavy metals such as chromium, manganese, molybdenum, and nickel are also associated with adverse health effects [13,14,17]. Based on available data, cadmium could be linked with the onset of lung cancer and poor prognoses [18]. Exposure to arsenic, mercury, lead, nickel, and chromium might be associated with breast, prostate, gastrointestinal, and gynecological cancers, while increased levels of chromium and molybdenum could be a risk factor for gallbladder cancer [19,20].

Keeping in mind everything mentioned above, the aims of this biomonitoring study were (1) to evaluate the urinary heavy metal(loid) (Cd, Pb, Hg, Cr, Mn, Mo, Ni, and As) contents in patients of both sexes with lung adenocarcinoma in the final stage (IIIB or IV); (2) to compare the levels of laboratory parameters, anthropometric measurements, and survival rates between patients with urinary heavy metal(loid)s below and above the limit of quantification; (3) to explore the association between heavy metal(loid) levels in urine and the laboratory results and anthropometric measurements; (4) to determine if cadmium and mercury urinary levels are related to exposure to tobacco smoking and amalgam dental fillings, respectively; and (5) to perceive exposure to possible sources of metal(loid)s in the context of their specific occupations, life habits, and living environments.

## 2. Materials and Methods

### 2.1. Patients

Over 90 patients, directed to the Institute for Lung Disease of Vojvodina, Sremska Kamenica, Serbia under the suspicion of lung cancer, were invited to participate in this study. After diagnostic procedures and pathohistological confirmation of lung cancer, 63 patients (36 male and 27 female) with inoperable stage IIIB and IV lung adenocarcinoma were enrolled in the study. The inclusion criteria were as follows: age > 18 years, creatinine clearance value >60 mL/min (calculated using the Cockroft–Gault formula), confirmed advanced stage (IIIB or IV) lung adenocarcinoma, and signed informed consent. All of the included patients had performance status ECOG 0 or 1. Pregnant patients, patients with a previous history of cancer, and diagnoses of other types of cancer, except adenocarcinoma with a lung cancer stage below IIIB or IV, were the exclusion criteria. All patients were included in the study from December 2021 to March 2022 while following the survival rate for a 24 month period, during which 48 of the 63 enrolled patients died. The enrolled patients were given a questionnaire regarding their occupations, smoking habits, and presence of amalgam dental fillings. The first morning, urine samples were collected within 24 h of hospitalization and before their specific oncological treatments.

Standard methods were applied for the following anthropometric measurements taken from all subjects included: weight, height, and waist and hip circumference. The body mass index (BMI) was calculated by dividing the body weight (in kg) by the square of the body height (in m). The waist-to-height ratio was obtained by dividing the waist circumference (in cm) by the height (in cm), while the waist-to-hip ratio was calculated as the ratio between the waist and hip circumferences in cm. The blood pressure was measured with a 65 mm sphygmomanometer in units of millimeters of mercury (mmHg). Blood samples were collected through antecubital venous punctures after 12 h of fasting. The alanine aminotransferase (ALT), aspartate aminotransferase (AST), γ-glutamyltransferase (GGT), lactate dehydrogenase (LDH), alkaline phosphatase (ALP), and C-reactive protein (CRP) contents were determined spectrophotometrically from the serum with a Cobas c311 automatic biochemical analyzer (Roche diagnostics). The complete blood count (CBC) with the differential was conducted with a Beckman Coulter, DxH 900 hematology analyzer.

### 2.2. Metal Analysis

#### 2.2.1. Chemicals

Standard solutions of Cr, Ni, Mo, and Cd were purchased from CPAchem (Stara Zagora, Bulgaria), Mn, As, and Pb solutions were purchased from AccuStandard Inc. (New Haven, CT, USA), and the Hg solution was purchased from Carlo Erba (Milan, Italy). Nitric acid (HNO_3_; 67–69%) for trace element analysis and hydrogen peroxide (H_2_O_2_; 30–32%) of a primer trace analysis grade were obtained from Fisher Chemical (Hampton, NH, USA). Ultrapure water was produced with a GenPure Water Purification System (Thermo Fisher Scientific, Langenselbold, Germany).

#### 2.2.2. Preparation of Urine Samples

Microwave digestion was performed using a Start D Microwave Digestion System (Milestone, Brondby, Denmark). A urine aliquot (1 mL) was placed in quartz inserts together with the 5 mL of nitric acid, while a mixture of 1 mL of hydrogen peroxide with 5 mL of deionized water was placed in teflon cuvettes. After the digestion and cooling, the mineralized samples were quantitatively transferred into graduated plastic tubes and diluted with deionized water to a total volume of 12 mL. A blank was prepared as urine using deionized water.

#### 2.2.3. Inductively Coupled Plasma Mass Spectrometry (ICP-MS) Analysis

The samples were analyzed using a 7700× ICP-MS spectrometer equipped with an integrated G3160B autosampler, Ni sample and skimmer cones, a MicroMist nebulizer with a PEEK connector, and a quartz spray chamber (Agilent Technology, Waldbronn, Germany). Analyses were performed under the operational conditions presented in Table S1. Either a “no gas” mode or a collision cell mode (He as the collision gas, or “He mode”) were used, with the latter used when needed to attenuate polyatomic interferences selectively. The monitored and reported isotope(s), analysis mode, and corresponding internal standard for each element are presented in Table S2. The quantification and method performance verification parameters are summarized in Table 1. Calibration curves covering concentrations ranging from 0.1 to 100 μg/L showed coefficients of determination (r^2^) greater than 0.998 for each element. The limit of detection and quantification (LOD and LOQ, respectively) were determined based on the standard deviation (SD) for reagent blanks measurements (*n* = 10; concentrations corresponding to 3 and 10 fold SD) (Table 1). Quality assurance was based on the analysis of certified reference materials (CRMs 1643f and TM-DSW.3), along with blanks, calibration verification standards, and duplicate analyses of random samples in analytical sequences. The applied method enabled determining the total Cr, total As, and total Hg.

## 3. Results

Sixty-three Caucasian patients of both sexes with inoperable stage IIIB or IV adenocarcinoma were enrolled in this study. The anthropometric characteristics, blood pressure, and laboratory results of the patients enrolled in this study are given in Table S3, together with the smoking habits of the enrolled patients and the presence of amalgam dental fillings.

Nine out of 36 male patients (25%) worked or had worked in land transport (three truck drivers, two tractor drivers, one tank driver, one dispatcher for a bus station, and two rail workers), and 11.11% (4/36) were construction workers, while other occupations included an orthodox priest, a judge, a baker, a waiter, a worker in a refinery, a carpenter, and a medical technician. Five of the 27 female patients were housewives, four were administrative workers, three worked or had worked in shoe factories, three were or had been salespersons, and the other occupations included a doctor, an economist, and a waitress.

In Table 2, the urinary concentrations of the observed metal(loid)s (Cd, Pb, Hg, Cr, Mn, Mo, As, and Ni) are given in μg/L, and for each element, the frequency of detection, range, 25th, 50th, 75th, and 95th percentiles, and median values along with the mean values and standard deviations are listed, as well as the mean or reference values defined by the ToxGuide of the US Agency for Toxic Substances and Disease Registry, followed by the percentage of participants exceeding the values defined by the ToxGuide [21]. Cadmium, mercury, and lead were quantified in 61.90%, 63.49% and 9.52%, of the analyzed samples, respectively. In 41.27% of the samples, chromium was detected above the LOQ, with the lowest measured value being 5.585 μg/L. Manganese was quantified in 31.75% of the enrolled patients, while nickel and molybdenum was quantified in 50.79% and 90.48% of the urine samples, respectively. The analyzed metalloid, As, was measured above the LOQ in 92.06% of the urine samples, with the lowest measured value being 1.246 μg/L.

The dilution was also taken into account, and the urinary levels of the observed metal(loid)s (Cd, Pb, Hg, Cr, Mn, Mo, As, and Ni in terms of μg/g creatinine (Cre) with the frequency of detection, the range, the 25th, 50th, 75th, and 95th percentiles, the median value, the mean value, and the standard deviation for each element, are summarized in Table 3. The differences in the urinary concentrations of the metal(loid)s in terms of μg/L and μg/gCre between the sexes among the enrolled patients are summarized in Table 4. The concentrations of urinary chromium (in μg/L) were statistically significantly higher in the male patients in comparison with the female patients (*p* = 0.035), but when expressed in μg/gCre, no differences were reported. Contrary to this finding, the levels of urinary cadmium (in μg/gCre) were higher in the female patients than in the male patients (*p* = 0.023). Sex differences were not obtained for other observed urinary metal(loid) concentrations.

The smoking habits are given in Table 5, followed by the cadmium urinary contents among the former smokers, current smokers, and those who never smoked, along with the Cd urinary levels for those with family histories of smoking and the family histories and urinary Cd contents of those who never smoked among the patients exposed and not exposed to second-hand smoking. No differences in the cadmium urinary levels were observed among patients with different smoking habits, between patients with families of smokers and those with no smokers in their families, or between those exposed to everyday second-hand smoking and those not exposed to it, indicating that smoking was not the main source of cadmium exposure. Table 6 presents a comparison between the Hg urinary concentrations in the patients with amalgam dental fillings and those without them. The levels of urinary Hg did not differ significantly among the patients with amalgam dental fillings and those who did not have amalgam dental fillings.

The anthropometric values, blood pressure, and laboratory findings were compared among patients with urinary metal(loid) concentrations above and below the LOQ using a Student’s *t*-test. The obtained results are summarized in Table S4 for chromium, manganese, nickel, and arsenic, while the comparison for molybdenum, cadmium, mercury, and lead is presented in Table S5. The ALT values were statistically higher in the patients with urinary Cr levels above the LOQ in comparison with the patients whose urinary Cr values were below the LOQ (Table S4). In the case of manganese (Table S4), the levels of eosinophiles had statistical differences between the patients with urinary Mn values above and below the LOQ. The waist-to-height ratio (WtHR) was higher in the patients with quantified As when compared with the patients with As levels below the LOQ (Table S4). Finally, as shown in Table S5, the mean corpuscular haemoglobin (MCH) and the mean corpuscular haemoglobin concentration (MCHC) were different statistically between patients with urinary Mo concentrations below and above the LOQ. As presented in Tables S4 and S5, none of the other examined parameters had statistically different values between patients with Cr, Mn, Ni, As, Mo, Cd, Hg, and Pb levels below and above the LOQ. The correlation between the observed parameters and the urinary metal(loid) concentrations (expressed in μg/L and μg/gCre) are given in Table S6 and Table S7, respectively. The chromium urinary levels were associated with the waist-to-hip ratio (WtHipR) (in μg/L; Table S6), as well as the CRP values (in μg/gCre; Table S7). The nickel urinary levels were correlated with the number of basophiles and the percentage of basophiles (Table S6). The molybdenum urinary levels (in μg/gCre) were correlated with the number of monocytes (Table S7), while the urinary cadmium levels were associated with the WtHipR and the number of red blood cells (RBCs) (Table S6). Finally, the mercury urinary levels were correlated with the number of white blood cells (WBCs) and neutrophiles, as well as the AST and GGT (in μg/L; Table S6). When observed in terms of μgHg/gCre, the mercury concentrations were associated with the number and percentage of monocytes, AST, ALT, and GGT (Table S7). The Kaplan–Meier survival analysis, based on the presence of metal(loid)s in the urine samples below and above the LOQ, is given in Table S8. There were no differences in the longest expected survival time between the patients who had any of the observed metal(loid)s quantified in their urine and those in which the analyzed metal(loid)s in their urine samples were below the LOQ.

All of the statistical analyses were conducted with IBM SPSS Statistics version 26, with the level of significance set to *p* < 0.05.

## 4. Discussion

This is the first time that the urinary levels of As, Ni, Cr, Cd, Hg, Pb, Mn, and Mo among stage IIIB or IV adenocarcinoma patients were reported. A short overview of each element and their association with lung cancer is given, followed by potential sources of exposure. The association between each element’s urinary level and lung cancer remains open.

### 4.1. Arsenic

The results in this research indicate that almost all included patients (58/63) had arsenic in their morning urine samples above the quantification limit (Table 2). Moreover, 44.44% (28/63) had urinary arsenic levels above 10 μg/L, and only one subject had a level above 100 μg/L. After dilution was taken into account, 58.73% (37/63) of the patients had detected arsenic levels above 10 μg/gCre in their morning urine samples (Table 3). There were no statistically significant differences in the urinary As levels between sexes (Table 4). It is worth noting that the reported values were much higher in comparison with the data reported in a Korean study where the urinary arsenic levels among Korean men varied between 0.36 μg/L and 36.79 μg/L, with a mean value of 7.63 μg/L [22], which is half of the mean value observed in this research (17.145 μg/L). Our findings corresponded better with the data obtained in a UK study among volunteers exposed to arsenic from private drinking water supplies, among whom the urinary arsenic levels reached as high as 426 μg/L, where all of them had measurable arsenic levels in their urine [23]. Several epidemiological studies confirmed increased lung cancer prevalence in the regions of Taiwan [24], Chile [25], and Argentina [26] due to high arsenic levels in drinking water or a high dietary intake, like in Japan [27]. There are inconsistencies in the published data regarding whether low-to-moderate arsenic concentrations in drinking water are associated with lung cancer [28,29]. In accordance with the evidence-based data, the EU Directive (98/83/EC), World Health Organization (WHO), and the Regulation on the Hygienic Acceptability of Potable Water (28/2019), the maximum level of arsenic in groundwater is 10 μg/L. Drinking water and food are recognized as sources of arsenic for the general population, but as an occupational contaminant, inhaled arsenic was reported as a risk factor for lung cancer morbidity and mortality. Cumulative inhaled inorganic arsenic exposure in professional environments (e.g., among miners and smelter workers, glass workers, and chemical workers) was associated with lung cancer. In addition, higher arsenic concentrations, inhaled over shorter periods, were found to be a greater risk for lung cancer development in comparison with lower concentrations over longer periods [30]. There are data which support arsenic’s genotoxic potential in primary human lung epithelial cells, resulting in tumor initiation due to chromosome damage and DNA double-strand breaks [31]. Furthermore, arsenic exposure induces reactive oxygen species (ROS) formation, which then disrupts the chromosome structure and stability [32]. Finally, arsenic impairs the DNA repair process, which allows proto-oncogene activation [33]. However, in our study, there were no statistically significant differences in any laboratory or biochemical parameters (Table S4) between patients with arsenic levels below and above the LOQ, nor was a correlation found with the arsenic urinary concentrations in μg/L (Table S6) or μg/gCre (Table S7) or arsenic levels above the LOQ and the survival time (Table S8). However, the patients with arsenic urinary concentrations above the LOQ had higher WtHRs in comparison with those below the LOQ of arsenic in their urinary samples. Due to the limited number of patients with As levels below the LOQ, this significance should be taken with limitations.

There was no evidence of occupational sources of exposure to arsenic in the enrolled patients, and thus possible environmental sources were considered. In the region of Vojvodina (northern Serbia), where this study was conducted, ground waters are endangered by arsenic (up to 0.750 mg/L). The concentration of arsenic in the drinking water was usually between 10 and 100 μg/L. The highest levels of arsenic in drinking water were measured in the city of Zrenjanin, with reports of arsenic concentrations in the drinking water as high as 450 μg/L [34,35]. The findings were in accordance with the obtained results from the Vojvodina region, indicating arsenic concentrations in about two thirds of the analyzed water samples above 10 μg/L, with 63% of all water samples exceeding Serbian and European standards for arsenic in drinking water [36]. The arsenic daily intake in Vojvodina in 2000 via food and nutrition was estimated to be as much as 60.9 ± 22.3 μg/day [34].

Air quality monitoring, conducted in urban areas of Vojvodina, indicated that the mean annual levels of arsenic were below the target value. However, for adults (16–70 years) exposed to arsenic and benzo(a)pyrene, the increased carcinogenic risk (ICR) was between 1 × 10^−6^ and 1 × 10^−4^ according to OEHHA guidance [37], taking into consideration the daily breathing rate, body weight, age sensitivity, and exposure duration. In addition, arsenic was estimated to have the highest carcinogenic risk in all age groups, being up to 1·10^−5^ for children and up to 4.9 × 10^−6^ for adults in each measuring site [38].

### 4.2. Chromium

In this research, 41.27% (26/63) of the patients had chromium in their morning urine samples above the LOQ, and all measured concentrations were above the mean values (0.22 μg/L) given by the ToxGuide (Table 2), indicating that the urinary chromium levels among our patients were well above the values expected for healthy adults [21]. The lowest measured value (5.585 μg/L) was more than 25 times above the mean level (0.22 μg/L) given by the ToxGude [21]. The results were also above the urinary chromium levels reported in Italy, with urinary concentrations from 0.05 to 1.27 μg/L and with 0.35 μg/L as the average value [39]. Moreover, the highest measured urinary chromium level in the research was up to 117.544 μg/L, which is 500 fold more than the mean value for healthy adults given by the ToxGuide [21]. The urinary Cr levels (in μg/L) were statistically significantly higher in the male patients in comparison with the female patients (*p* = 0.035, Table 4), while when expressed in μg/gCre, the chromium levels remained higher in the male group, although they were not statistically significant.

Occupational exposure to chromium was associated not only with the increased incidence of lung and nasal cancers as well as buccal cavity and pharynx cancers but also enhanced risk of overall mortality due to lung, larynx, bladder, kidney, testicular, bone, or thyroid cancer [40]. A great number of evidence-based data suggest that direct chromium carcinogenicity is associated with lung cancer development and progression [41,42,43]. The mechanisms of chromium toxicity and carcinogenicity are not completely understood since chromium (VI) is unreactive toward most biomolecules, and the impact of Cr(VI) on the cellular stress response could have a central role in its adverse effects. Moreover, due to chromium’s intracellular reduction, chemically versatile species are generated and may attribute to its carcinogenicity [44]. In this study, the urinary chromium levels could not be associated with the expected survival rate (Table S8), but the possible harmful effects were observed as statistically significantly higher ALT levels in the patients with urinary chromium levels above the LOQ in comparison with those having values below the LOQ (Table S4). In addition, there were statistically significant correlations between the urinary chromium levels (in μg/L) and WtHipR (Table S6) and between the urinary chromium levels (in μg/gCre) and CRP (Table S7).

The highest concentration of chromium was detected in a retired butcher, thus excluding the possibility for occupational exposure. The possible sources of Cr are foods, due to contaminated soil. Chromium, together with nickel and cobalt, was detected in the soil in Vojvodina [45] and in the sediments of the drainage channels as well [46]. Moderately strong soil enrichment with chromium salts was estimated based on the levels of chromium measured in samples near the leather industry in Vojvodina [47], which is in accordance with the increased chromium levels found near leather and cement plants located in Vojvodina [48]. The presence of chromium was confirmed in vegetables in the Vojvodina Province, with the highest content in parsley [49], while another study indicated spinach as the largest chromium accumulator [50], although the levels of chromium were below the maximum permissible levels recommended by the Food and Agriculture Organization (FAO) and World Health Organization (WHO). Nevertheless, chromium was detected in the goat meat of male Saanen goat kids from Vojvodina [51], indicating the presence of chromium in animal food as well as the bioaccumulation process of chromium in the food chain.

### 4.3. Nickel

Nickel was detected in 50.79% (32/63) of the urine samples from the observed patients. It should be emphasized that in all samples with Ni urinary levels above the LOQ, the urinary values were actually above the urinary reference range (1–3 μg/L) for healthy adults according to the ToxGuide [21], and the concentrations were as high as 446.50 μg/L (Table 2). No statistically significantly different urinary Ni concentrations were recorded between the sexes (Table 4). The urinary nickel concentrations among the lung cancer patients enrolled in this study were above the urinary nickel levels detected in France, where nickel was detected in 98.38% of the urine samples but with a geometric mean of 2.00 μg/L [52]. Moreover, the nickel concentrations in the examined urine samples, when expressed in terms of creatinine, reached a median nickel concentration of 170.275 μg/gCre (Table 3), which also surpassed the observed levels in Belgium, where the median nickel concentration was only 1.79 μg/gCre [53].

Over the years, several epidemiological studies have confirmed that high-concentration exposure to nickel is unequivocally associated with lung cancer development, but low nickel concentration chronic exposure was also confirmed to have carcinogenic potential [54]. Nevertheless, exposure to nickel compounds, even below the occupational exposure limit, enhances the risk of lung cancer and can be associated with a risk of cancer development at other body sites [55]. Even water-soluble nickel salts were observed as a risk factor for both lung and nasal cancer, despite the contradicting epidemiological evidence [56]. Elemental nickel is classified as a suspected human carcinogen (CLP), or group 2B according to the International Agency for Research on Cancer (IARC), while nickel compounds (nickel sulphate, a combination of nickel sulphides and oxides) are listed in group 1, being carcinogenic to humans.

Although the exact mechanisms of Ni carcinogenicity are unclear, the DNA damage caused by Ni and Ni compounds and repressing of the DNA repair mechanism are presumed to be the main Ni carcinogen effect [57]. In this study, the presence of nickel above the LOQ in the urine samples did not affect the estimated survival rate (Table S8). The comparison of the anthropometric and laboratory parameters showed no differences within the patients who had urinary nickel levels below and above the LOQ (Table S4). Although the correlations of all observed parameters with urinary Ni concentrations in μg/gCre indicated no statistical significance (Table S7), the levels of basophils both as absolute and relative values were associated with the nickel urinary levels (in μg/L) as given in Table S6. The possible influence of nickel on the production of a specific white cell line is yet to be understood. The highest nickel values were detected in a sound production mixer who was reported to be working with batteries. Keeping in mind that none of the other patients could be exposed to nickel due to professional reasons, other sources such as air, soil, and food were observed. The air quality analysis of the urban areas in Vojvodina confirmed that the nickel levels did not exceed the limit values [38]. Nickel was detected in the soil in Vojvodina together with chromium as mentioned [45]. In addition, the average Ni content was above the maximum allowable concentration (MAC) defined by the Serbian soil quality standard [58]. Higher Ni values were also found in an illegal landfill in Vojvodina [59]. Nickel contents higher than the MAC in nine samples from Fruška Gora mountain (Vojvodina) was attributed to a geochemical origin [60]. Another study estimated moderate and moderately strong soil enrichment with Ni in samples collected in Vojvodina from locations near leather and cement factories [47]. The higher nickel content in the alluvial-diluvial soils of the region of Srem (Vojvodina, Serbia) was attributed to alluvial processes which transported nickel from Fruška Gora mountain, while the maximum level was measured near a cement plant and leather factory [48]. Regardless of its origin, nickel from the soil enters biological species and thus gets into the food chain. Together with chromium, the highest nickel concentration was measured in parsley leaves due to bioaccumulation from contaminated soil [49]. In addition, in another study, nickel was found with the highest average content in spinach leaves, broccoli, and tomatoes in Vojvodina [50]. Nickel enters the food chain, and its accumulation in the muscles or livers of animals can be expected. In accordance with this hypothesis, the nickel levels in the muscles and livers of pig genetic lines produced in Vojvodina were higher than the levels measured in pork in some developed countries [61,62]. It is worth mentioning that nickel was detected even in sunflower honey in Serbia [63]. Hence, non-occupational exposure to both nickel and chromium among the general population in this region is possible. The high exposure rates to chromium and nickel reported in this study among lung carcinoma patients require further follow-up and observing urinary chromium and nickel as potential biomarkers. Significantly elevated Cr and Ni contents in lung tumors among lung cancer patients in comparison with normal lung tissue from noncancerous controls have already been reported [64]. However, there is a lack of epidemiological data that associate nickel compounds absorbed after oral administration with cancer development. Studies conducted on animals which link the dietary application of nickel compounds with cancer initiation are scarce as well. The CONTAM Panel concluded that dietary nickel administration is unlikely to result in cancer in humans [65].

### 4.4. Cadmium

Cadmium was detected in 61.90% (39/63) of the patients, with a mean value of 5.524 μg/gCre (Table 3) in this research. In all patients with urinary Cd levels above the LOQ, the urinary Cd concentration was above 0.195 μg/gCre, which is recognized as the mean value for healthy adults by the ToxGuide [21]. Based on a meta-analysis conducted on over 20,000 participants, the relative risk for lung cancer was estimated for cadmium urinary levels between 1.21 and 1.70 μg/gCre, while the pooled relative risk amounted to 1.68 μg/gCre (*p* < 0.0001) [66]. The Cd urinary values in this study were above the estimated relative risk, amounting to 1.68 μg/gCre [66] even in the first quartile of the enrolled patients. The highest cadmium urinary level measured among our lung cancer patients reached 28.356 μg/gCre. The data obtained in this study put females at higher risk, given that the urinary Cd concentration levels were significantly higher among the women in comparison with the male lung adenocarcinoma patients (*p* = 0.023, Table 4). However, the estimated survival rate was not affected by the presence of Cd in the urine samples (Table S8), nor was any observed parameter among the patients with Cd urinary concentrations above the LOQ statistically different when compared with those with levels below the LOQ (Table S5). The correlation between the anthropometric and laboratory findings with Cd urinary concentrations expressed in μg/gCre (Table S7) were without statistical significance, while those expressed in μg/L were associated with the WtHipR (Table S6). Given the fact that chromium urinary levels were also associated with the WtHipR, this anthropometric parameter could be taken into consideration for further analysis.

When there are no unusually prominent environmental or occupational sources, the common urinary cadmium concentration should be <2 μg Cd/gCre in the Western population [67], which corresponds to the cadmium levels of the first quartile reported in this paper. Cadmium has been classified as a carcinogen by the IARC, and it is listed as a group I carcinogen. Both occupational and environmental cadmium exposure have been associated with lung cancer [68]. Cadmium carcinogenicity is attributed to the production of ROS and alteration of the DNA repair mechanism which results in cell resistance to apoptosis [69]. In addition, cadmium blood levels were observed as a potential biomarker for early diagnosis of lung cancer among former smokers. Namely, the odds ratio for patients with lung cancer in the highest quartile of cadmium levels versus the lowest one was fourfold (OR = 4.41, *p* < 0.01) in former smokers but not in current smokers or those who never smoked. The association of cadmium blood levels among former smokers and lung cancer incidence is present in both early- and late-stage lung cancers [70]. The urinary cadmium concentration is recognized as a biomarker for lung cancer risk, and even low cadmium concentrations in urine samples has been associated with a risk for total cancer and lung cancer. Interestingly, the urinary cadmium levels among the enrolled patients could not be associated with smoking habits. There were no significant statistical differences in the cadmium levels among the former smokers, current smokers, and those who never smoked (Table 5). In addition, the cadmium levels could not be associated with second-hand smoking (Table 5). The small sample size is the main limitation for making more valid conclusions. Nevertheless, the study conducted on stage I–IV lung cancer patients could not also link cadmium blood levels with lung cancer incidence among the current smokers and those who never smoked [70]. It was assumed that the patients changed their smoking habits shortly after diagnosis. In accordance with this assumption, seven of the enrolled patients reported smoking cessation in the past six months. Regardless of smoking, the high urinary cadmium levels indicated possible environmental exposure, since none of the patients could be associated with an occupational risk. Cadmium has been detected in the vineyard soil in Vojvodina [60]. Although the cadmium concentrations in rabbit livers from 17 different locations in Vojvodina were below the level permitted by Serbian regulations, the cadmium levels exceeded the maximum established values in the samples from two locations [71]. These results are in accordance with recent findings which confirmed detectable cadmium values below the MAC in superficial waters in Vojvodina as well as in the whole bodies of fish. However, Cd concentrations above the permitted levels were measured in the kidneys and livers of wild animals (rabbit and roe deer) [72]. Cd accumulation in the kidneys was confirmed in other animals as well, since the concentration of cadmium in the kidneys of female cattle from dairy farms and swallow belly Mangulica pigs in Vojvodina were below the level permitted by Serbian and EU regulations but were higher in comparison with the amount measured in their livers [73,74]. Higher average cadmium levels in pork livers from 10 different genetic lines from Vojvodina in comparison with pork livers from developed EU countries were also reported [75]. A study conducted 30 years ago in this region gave unequivocal confirmation of the Cd pathways in the food chain. Cd was detected in atmospheric deposits in the area of the town of Kikinda, Vojvodina due to the construction and metal processing industries. After three-year economic sanctions in Serbia, the decrease in Cd levels in the Kikinda area in atmospheric deposits resulted in a decrement in Cd concentrations not only in the soil but also in cattle feed and milk [76]. On the other hand, a recent study indicated moderate soil pollution in Vojvodina, with Cd in the surface layer. The highest Cd value was detected in agricultural soil located between a pesticide factory and a building material factory. Fertilizers applied in agricultural areas and fossil fuels combusted in different processes were suspected as the main sources of anthropogenic Cd [47].

### 4.5. Mercury

In our study, mercury was detected in 63.49% of the patients’ urine samples (40/63), where the lowest detected value (1.94 μg/L) was more than 10 times above the median urinary value (0.140 μg/L) for healthy adults given by the ToxGuide [21], as shown in Table 2. Furthermore, all patients with measured urinary mercury levels in this study (above 1.9 μg/L) exceeded the 95th percentile urinary Hg value (1.8 μg/L) for the USA [77]. No adverse effects could be expected at levels below the 5 μg/gCre or 7 μg/L urinary levels [78]. Taking into account this reference value, 47.62% (30/63) of the observed patients surpassed 5 μg/gCre versus 2.3% among the Canadian population, and 20.63% (13/63) exceeded the 7 μg/L urinary level, in comparison with only 1.8% of the Canadian population [78]. In addition, the mean value (7.237 μg/L) of the patients in this study was more than double compared with the mean value of 3.09 μg/L obtained among US male participants [79]. No differences were observed among the sexes (Table 4). In addition, mercury had no effect on the estimated survival rate reported in this study (Table S8), and no statistically significant differences in the observed parameters between patients with urinary mercury levels below or above the LOQ were reported (Table S5). However, it has been reported that mercury elicits a prooxidative state in the cells and stimulates proinflammatory cytokine production, thus promoting their proliferation [80]. In this study, urinary Hg concentrations (in μg/L) were associated with the total number of WBCs and the number of neutrophils (Table S6). Meanwhile, when the Hg urinary concentration was expressed in μg/gCre, it was associated with the total and relative number of monocytes (Table S7). Moreover, the potential toxic effects of high Hg levels were observed through the positive association between the μgHg/L amount and the AST and GGT serum levels (Table S6), as well as between the μgHg/gCre amount and the ALT, AST, and GGT values (Table S7). Dental fillings could not be observed as a source of mercury since no statistically significant difference was observed in the urinary mercury content between the patients with dental amalgam fillings and those without them (Table 6).

To the best of our knowledge, there is only one study that associated mercury and lung cancer mortality, according to which the hazard death ratio from all causes was 1.55 (*p* = 0.04) for mercury in lung cancer patients when the highest quartile was compared to the lowest one [81]. The occupational exposure to mercury among mercury workers in Slovenia and Ukraine was not confirmed as an occupational risk factor, although the mortality rate increased since the workers were exposed to radon and crystalline silica as well [82]. Since none of the enrolled patients were professionally exposed, environmental pollution may once again be assumed as a source of mercury. In the Mediterranean region, between 1983 and 1998, mercury emissions were elevated by 39% mainly due to waste incineration [83]. Mercury was detected in the Vojvodina agricultural soil, with the highest concentration in the alluvial plains, suggesting anthropogenic Hg sources near rivers [84]. Higher Hg values were registered in agricultural soil samples next to cellulose industries and sugar refineries. The soil samples from Vojvodina were classified as moderately polluted with Hg due to various anthropogenic sources such as agrochemicals, urban environment activities, and fossil fuel combustion [47]. The mercury concentrations in 2019 in surface water exceeded the maximum permitted value of 0.07 μg/L, although it should be stressed that the Hg concentrations in both superficial water and whole bodies of fish, as well as in the kidneys and livers of wild animals (rabbit and roe deer), did not exceed the permitted limits [72]. Marine fish, freshwater fish, and canned fish on the Serbian market contained mercury below the maximum level defined by the European Union and Serbian regulations. The estimated weekly intake based on the mean mercury value in fish and an average body mass of 70 kg was 0.095 μg/kg b.w./week, which is lower than the safe limit. However, the highest mercury content was reported in canned fish [85]. Mercury was found in female cattle in Vojvodina in both the liver and the kidneys, with higher concentrations detected in the liver [86]. Hence, the food chain could be one of the possible sources of mercury in the lung patients of Vojvodina, but the sources of high Hg values still have yet to be determined.

### 4.6. Lead

In our research, six patients had Pb in their urine samples above the LOQ (12 μg/L), as shown in Table 2, with no differences between the sexes (Table 4), which is more than 10 times above the highest quartile (>1.26 μg/L) reported in a study by Li et al. [87]. Lead and inorganic lead compounds, according to the IARC, are recognized as possibly carcinogenic to humans (Group 2B). Direct DNA damage, inhibition of DNA synthesis or repair, formation of ROS, and oxidative damage to DNA are the possible mechanisms of lead carcinogenicity [88]. There are contradicting epidemiological data regarding the association of lead with lung cancer. A study conducted almost 30 years ago on over 20,000 people professionally exposed to lead suggested lead as a risk factor for lung cancer. The risk was evaluated as a 1.4-fold increase in the overall cancer incidence among the group exposed to lead and a 1.8-fold increase in the incidence of lung cancer among the group with high exposure to lead (blood levels over 1.0 μmol/L). It should be emphasized that the observed subjects were co-exposed to engine exhaust [89]. On the contrary, more recent studies indicated that exposure to lead compounds presents no increased risk of lung cancer [90]. Despite the discrepancies reported in previous studies, urinary lead levels were associated with cancer-specific mortality. The highest quartile included participants with high urinary lead levels (>1.26 μg/L), who were more likely to be males older than 70 years. In addition, urinary lead levels were correlated with all-cause mortality [87]. The small number of patients with detected urinary lead levels above the LOQ may be the single most important reason for no statistical significance of lead levels for any of the observed parameters (Tables S5–S7) or the estimated survival time (Table S8) in this study. A high urinary lead level could be attributed to occupational exposure in only one patient, since the patient was a professional driver. However, the results revealed that 25% (9/36) of the male patients were chronically exposed to diesel exhaust at their workplaces, thus confirming the association between lung cancer and diesel exhaust. Diesel exhaust was classified by the IARC in 2012 as a group 1 carcinogen in humans based on evidence of its carcinogenicity to the lung [91]. Other possible sources of exposure to lead include water, soil, and food. Lead was detected in slightly elevated levels in Vojvodina soil in its more mobile form in the surface layer [48]. The bioaccumulation of lead from soil and water in Vojvodina was confirmed, since the average Pb levels in potatoes and carrots exceeded the maximum concentrations established by EU and Serbian regulations [58]. These findings are in accordance with recent research where 196 hare liver samples from 17 different locations were analyzed, and high lead levels were detected in the majority of the samples [71]. Based on the measured lead concentrations in different matrices, humans might be exposed to lead via multiple sources in this region. Hence, there is a high probability of low lead concentrations in urine samples (<12 μg/L). ICP-MS, as the state-of-the-art analytical approach, was applied and encouraged the understanding of the importance of lead as a risk factor for the various health outcomes in this research. Since acceptable (“safe”) exposure levels to lead are not defined, improvements in quantification remain a big challenge.

### 4.7. Molybdenum

It should be noted that the median levels for molybdenum among our female patients (67.829 μg/gCre) were lower in comparison with the data reported among women with breast cancer (85.20 μg/gCre) [92]. On the other hand, the mean urinary molybdenum levels were as high as 55.2 μg/gCre among pregnant women in Mexico due to increased intake of hot peppers [93]. The highest measured values were up to 350 μgMo/gCre in this study. Additionally, almost 50% of the patients had Mo urinary levels above the mean value (32 μg/L) for healthy adults according to the ToxGuide [21], as presented in Table 2, with no differences between the sexes (Table 4). The presence of molybdenum above the LOQ in the urine samples affected the red cell line, with lower MCH and MCHC levels in comparison with those with molybdenum values below the LOQ (Table S5), followed by an association of urinary Mo levels with the monocyte count (Table S7) and no effect on the estimated survival time (Table S8). Due to a small sample size, all obtained statistically significant differences in this study and any associations should be taken with precaution.

The data regarding molybdenum’s association with cancer are conflicting. Lung cancer was associated with extremely high inhaled molybdenum trioxide levels in mice. Thus, molybdenum trioxide is considered to be possibly carcinogenic to humans according to the IARC. On the other hand, molybdenum is a vital trace element stored in the bones, glands, liver, and kidneys which is mainly excreted through urine. Molybdenum compounds are applied as anticancer agents in the therapy of esophageal cancer and breast cancer [94]. The epidemiological data regarding the molybdenum content and cancer are also contradictory. The molybdenum content in toenails was associated with reduced incidence of breast cancer overall [95]. On the contrary, high urinary molybdenum concentrations were associated with increased risk of breast cancer among postmenopausal women [92]. Having in mind the high levels of urinary Mo (urinary Mo levels above 100 μg Mo/gCre) obtained in this study, not only dietary but also other sources of exposure should be considered. To the best of our knowledge, there are no data regarding the possible contamination of soil or surface water with molybdenum in the Vojvodina region. Molybdenum has not been the focus of studies as a possible anthropological pollutant in this area. However, high urinary molybdenum levels should be acknowledged and observed among cancer patients. Usually, mining sites are recognized as sources of molybdenum, and thus lake reservoirs downstream of mining areas and their mobility to stream waters was a possible route of molybdenum enrichment of soil and water [96,97].

### 4.8. Manganese

In this study, 9.52% (6/63) of the lung cancer patients had urinary manganese concentrations above 8 μg/L, the upper reference value for healthy adults given by the ToxGuide [21], as presented in Table 2. There were no differences in the urinary manganese levels between the male and female patients (Table 4). The presence of manganese above the LOQ did not affect the estimated survival rate (Table S8), nor could the urinary Mn levels be associated with any given parameters (Tables S6 and S7). However, the absolute and relative levels of eosinophils were lower in the patients with urinary Mn values above the LOQ in comparison with those with levels below the LOQ (Table S4). These findings are in accordance with the previously reported high Mn intake association with decreased eosinophils [98]. Manganese is a cofactor and an antioxidant necessary for blood formation and cell metabolism concentrated mainly in the bones, brain, liver, pancreas, and kidneys. The functioning of the musculoskeletal and immune systems is dependent on the manganese content as well [99]. Manganese’s association with cancer is contradictory. The recently published data indicate that manganese inhibits the viability of prostate cancer cells and the viability of myelogenous leukemia K562 cancer cells [100,101]. Manganese was recognized as a crucial factor in antitumor immune responses, which contributed to the efficacy of clinical immunotherapy [102]. Manganese deficiency was associated with breast cancer [103], while manganese superoxide dismutase was recognized as a key target in cancer prevention [104]. Conversely, a high manganese content in drinking water in China was correlated with cancer incidence and mortality [105]. In addition, a positive association was found between blood manganese levels and liver stiffness among patients with chronic obstructive pulmonary disease [106]. Manganese is excreted through urine in rather small amounts. The high manganese levels observed in this study could be the result of over consumption of manganese-rich food or over-supplementation on one hand or environmental exposure on the other. The high levels of Mn in the water system and sediments enriched due to mining and processing plants confirm that manganese can also be an environmental pollutant [107,108]. There is a lack of data about potential manganese pollution in Vojvodina, and practically no research has been carried out regarding high manganese levels being associated with lung cancer. This is the first time that in 9.52% of lung cancer patients, extremely high urinary manganese levels were reported.

### 4.9. Strengths and Limitations

The obtained results are preliminary, with its limited sample size as the main limitation. In addition, all of the enrolled patients were from the same geographical region. The ICP-MS method applied in the present study for quantification of heavy metal concentrations in the urine samples is a “state-of-the-art” technique, and only urinary concentrations of heavy metal(loid)s above the LOQ were discussed. However, this approach resulted in lower frequency of detection for lead and nickel despite their ubiquitous presence in different matrices. It could be assumed that lower concentrations of the aforementioned heavy metals could be quantified by enhancing the sensitivity of the method.

On the other hand, urinary metabolomics in advanced cancer patients is an innovative approach in the discovery of cancer biomarkers and might open the door to early cancer screening. To the best of our knowledge this is a unique study on heavy metal(loid) urinary levels among a specific group of Caucasian patients of both sexes with exclusively one lung cancer subtype (adenocarcinoma) which was diagnosed practically in its terminal stage. The importance of everyday exposure to low doses of a single heavy metal(loid) or their combination over long periods to cancer development and the severity of the diagnosed cancer, along with the significance of urine metal(loid) levels for cancer detection and progression rates, are challenges which have yet to be addressed. Therefore, continuous monitoring of air, water, soil, and food quality is necessary in order to develop preventive measures for the reduction of human exposure to heavy metal(loid)s and other compounds with carcinogenic potential.

## 5. Conclusions

Eight metal(loid) urinary concentrations were quantified in patients with non-operable, terminal lung adenocarcinoma in this study. Carcinogenic metal cadmium was quantified in over 60% of the samples, with higher concentrations in the female patients regardless of smoking habits. Arsenic was quantified above 10 μg/L in more than 40% of the samples, and lead was measured in almost 10% of the samples. Mercury was quantified in more than 60% of the samples without association with amalgam dental fillings and correlated positively with the AST, ALT and GGT levels. Urinary chromium concentrations were higher in the male patients and associated with the incrementing of CRP serum levels, while nickel was quantified above the reference values for healthy adults in over half of the participants. High urinary levels of manganese, particularly above the reference values for healthy adults, were measured in over 30% of the enrolled patients, while in over 40% of the samples, the levels of molybdenum exceeded the mean reference value for healthy adults. None of the metal(loid)s were related to the expected survival rate of the patients. The obtained results suggest that lung cancer patients are ubiquitously exposed to different heavy metal(loid)s. Further studies are indispensable to have deeper insight into metal(loid) exposure’s association with the development, progression, and survival prediction of lung adenocarcinoma patients.

## Figures and Tables

**Table 1:** ICP-MS quantification and method performance verification parameters.

Element ^a^	Symbol	LOD (μg/L Urine)	LOQ (μg/L Urine)	Accuracy (%) ^b,c^	Repeatability (%RSD)	Reproducibility (%RSD)
Chromium	Cr	1.6	4.8	99.6	2.97	3.42
Manganese	Mn	0.8	2.4	102.9	3.44	6.00
Nickel	Ni	8	24	100.1	4.09	7.99
Arsenic	As	0.4	1.2	101.1	1.34	2.35
Molybdenum	Mo	2	6	103.7	1.78	3.65
Cadmium	Cd	0.17	0.5	93.7	1.59	2.34
Mercury	Hg	0.63	1.9	105.2	1.11	2.85
Lead	Pb	4	12	92.8	4.45	9.01

^a^ The mixed standard solutions were prepared by mixing and diluting individual stock standard solutions of elements (1 g/L) with ultrapure water and nitric acid. Standard solutions of Cr, Ni, Mo, and Cd were purchased from CPAchem (Stara Zagora, Bulgaria), Mn, As, and Pb solutions were purchased from AccuStandard Inc. (New Haven, CT, USA), and Hg solution was purchased from Carlo Erba (Milan, Italy). ^b^ Standard reference material (CRM) 1643f: trace elements in water from the National Institute of Standards and Technology (Gaithersburg, MD, USA). ^c^ Standard reference material (CRM) 1642d: mercury in water from the National Institute of Standards and Technology (Gaithersburg, MD, USA).

**Table 2:** Urinary concentrations of the observed heavy metals and metalloids in μg/L, the frequency of detection, mean and median values with standard deviation, the range (minimum and maximum), and the 25th, 50th, 75th, and 95th percentiles together with the mean or reference values defined by the ToxGuide of the US Agency for Toxic Substances and Disease Registry, followed by the percentage of participants exceeding the values defined by the ToxGuide.

		Cr (μg/L)	Mn (μg/L)	Ni (μg/L)	As (μg/L)	Mo (μg/L)	Cd (μg/L)	Hg (μg/L)	Pb (μg/L)
N	≥LOQ	26	20	32	58	57	39	40	6
	<LOQ	37	43	31	5	6	24	23	57
Frequency of detection		41.27% (26/63)	31.75% (20/63)	50.79% (32/63)	92.06% (58/63)	90.48% (57/63)	61.90% (39/63)	63.49% (40/63)	9.52% (6/63)
Mean		25.276	6.312	98.745	17.145	48.478	2.720	7.237	15.681
Median		11.290	5.244	59.624	9.406	32.672	2.266	5.878	14.893
Standard deviation		28.855	3.560	91.391	21.001	55.110	2.014	5.505	2.596
Minimum		5.585	2.782	26.020	1.246	6.463	0.617	1.940	12.828
Maximum		117.544	14.989	446.498	119.405	350.696	11.828	28.115	19.340
Percentile	25	7.425	3.495	37.160	3.881	19.740	1.233	3.697	13.529
	50	11.290	5.245	59.624	9.406	32.672	2.266	5.878	14.893
	75	27.895	8.582	133.616	23.591	50.641	3.782	8.661	18.614
	95	106.291	14.886	339.827	62.772	147.117	5.667	21.931	n.a.
ToxGuide mean levels		0.22 μg/L	1.19 μg/L	n.a.	n.a.	32 μg/L	0.185 μg/L	n.a.	n.a.
ToxGuide median levels		n.a.	n.a.	n.a.	n.a.	n.a.	n.a.	0.140 μg/L	n.a.
ToxGuide reference levels		n.a.	1–8 μg/L	1–3 μg/L	<100 μg/L	n.a.	n.a.	n.a.	n.a.
Frequency above ToxGuide levels		41.27% (26/63)	9.52% (6/63)	50.79% (32/63)	1.58% (1/63)	46.03% (29/63)	61.90% (39/63)	63.49% (40/63)	n.a.

n.a. = not applicable since there were no ToxGuide levels.

**Table 3:** The urinary levels of the observed heavy metals and metalloids in terms of μg/g creatinine (Cre), with the frequency of detection, the range, the 25th, 50th, 75th, and 95th percentiles, the median value, the mean value, and the standard deviation for each element.

		Cr (μg/gCre)	Mn (μg/gCre)	Ni (μg/gCre)	As (μg/gCre)	Mo (μg/gCre)	Cd (μg/gCre)	Hg (μg/gCre)	Pb (μg/gCre)
N	≥LOQ	26	20	32	58	57	39	40	6
	<LOQ	37	43	31	5	6	24	23	57
Mean		72.216	16.471	240.715	31.927	79.262	5.524	15.330	47.992
Median		27.305	12.230	170.275	16.714	55.108	3.506	6.479	54.246
Standard deviation		118.607	14.218	272.106	41.694	92.450	5.257	31.630	29.300
Minimum		2.812	2.040	12.098	1.155	6.674	0.481	0.752	6.190
Maximum		555.067	54.312	1212.742	237.399	537.641	28.356	197.619	90.141
Percentiles	25	11.400	5.118	52.992	6.001	27.498	2.531	4.614	19.761
	50	27.305	12.230	170.276	16.714	55.108	3.506	6.479	54.246
	75	79.414	22.690	246.476	38.624	91.760	7.078	10.579	66.668
	95	466.498	53.628	947.892	104.598	351.444	16.666	48.072	n.a.

n.a. = not applicable due to small number of patients with lead levels ≥LOQ.

**Table 4:** The differences in the urinary concentrations of the metal(loid)s in μg/L and μg/g creatinine between the sexes (M = male; F = female) among the enrolled patients.

	Gender	N ≥ LOQ	Mean (μg/L)	*p* Value	Mean (μg/gCre)	*p* Value
Cr	M	14	36.154 ± 35.3357	0.035 *	101.324 ± 155.039	0.161
	F	12	12.586 ± 9.505		38.258 ± 35.040	
Mn	M	8	7.236 ± 3.496	0.356	18.784 ± 19.049	0.567
	F	12	5.697 ± 3.616		14.929 ± 10.573	
Ni	M	17	86.041 ± 103.351	0.403	173.359 ± 280.119	0.138
	F	15	113.143 ± 76.609		317.052 ± 250.039	
As	M	33	17.890 ± 23.585	0.750	23.504 ± 25.719	0.110
	F	25	16.162 ± 17.436		43.047 ± 54.958	
Mo	M	32	52.059 ± 62.906	0.567	59.343 ± 68.306	0.065
	F	25	43.893 ± 44.003		104.759 ± 112.740	
Cd	M	22	2.905 ± 2.379	0.495	3.692 ± 2.981	0.023 *
	F	17	2.480 ± 1.448		7.894 ± 6.587	
Hg	M	24	7.837 ± 5.966	0.385	10.325 ± 13.095	0.225
	F	16	6.339 ± 4.772		22.837 ± 47.308	
Pb	M	1	14.887	n.a.	90.141	n.a.
	F	5	15.840 ± 2.869		39.562 ± 23.240	

* Statistically significant difference *p* < 0.05, n.a. = not applicable.

**Table 5:** Differences in cadmium levels in μg/gCre and μg/L based on smoking habits, origin from family of smokers or those who never smoked, and exposure to everyday smoking. A comparison of the pack-year and the age they started to smoke is given to emphasize the lack of statistical difference of these variables between former and current smokers.

	Former Smokers (29/63)	Current Smokers (29/63)	Never Smoked (5/63)	*p* Value	Family of Smokers (28/39)	Family Never Smoked (11/39)	*p* Value	Everyday Second-Hand Smoking (21/39)	Rare or No Second-Hand Smoking (18/39)	*p* Value
	Mean ± SD	Mean ± SD			Mean ± SD	Mean ± SD		Mean ± SD	Mean ± SD	
Pack-year	46.93 ± 25.85	52.62 ± 24.18	-	0.395	/	/		/	/	
Started to smoke	20.28 ± 6.01	18.24 ± 4.19	-	0.689	/	/		/	/	
Cd (μg/gCre)	4.569 ± 4.044	6.568 ± 6.158	1.827 *	0.581	6.208 ± 5.914	4.180 ± 3.078	0.290	6.105 ± 5.873	4.845 ± 4.506	0.463
Cd (μg/L)	2.681 ± 1.188	2.792 ± 2.611	1.971 *	0.922	2.964 ± 2.232	2.189 ± 1.394	0.294	2.721 ± 1.590	2.718 ± 2.469	0.997

* Only in one patient who was never a smoker were the levels of urinary Cd above the LOQ.

**Table 6:** The difference in mercury urinary levels (in μg/gCre and μg/L) between the participants who reported amalgam dental fillings and those without them.

	Amalgam Dental Fillings	No Amalgam Dental Fillings	*p* Value
	**Mean ± SD**	**Mean ± SD**	
Hg (μg/gCre)	11.420 ± 12.264	18.260 ± 39.749	0.523
Hg (μg/L)	6.519 ± 6.479	7.857 ± 4.937	0.470

## Data Availability

The datasets used and analyzed in the current study are available from the corresponding author on reasonable request.

## Supplementary Materials

The following supporting information can be downloaded at https://www.mdpi.com/article/10.3390/toxics12070490/s1. Table S1: Sample preparation and ICP-MS instrument operating conditions; Table S2: ICP-MS monitored and reported isotope(s), analysis mode and corresponding internal standard for each element; Table S3: The anthropometric characteristics, blood pressure and laboratory findings of the enrolled patients presented as mean values with standard deviation, as well as smoking habits, belonging into family of smokers, exposure to second-hand smoking and presence of amalgam dental fillings given as percentage; Table S4: The difference in anthropometric characteristics, blood pressure and laboratory findings of the patients with urinary chromium, manganese, nickel and arsenic concentration above and below LOQ; Table S5: The difference in anthropometric characteristics, blood pressure and laboratory findings of the patients with urinary molybdenum, cadmium, mercury and lead concentration above and below LOQ; Table S6: The statistical parameters of the correlations between the urinary metal(loid)s concentration in μg/L and anthropometric characteristics, blood pressure and laboratory findings; Table S7: The statistical parameters of the correlations between the urinary metal(oid)s concentration in μg/gCre and anthropometric characteristics, blood pressure and laboratory findings; Table S8: Estimate of the longest survival time ± standard error based on the Kaplan-Meier survival analysis upon the presence of each metal(oid) in the urine samples below or above LOQ values.
