# Supplementary material for: Biomonitoring Study of Toxic Metal(loid)s: Levels in Lung Adenocarcinoma Patients
Source: Toxics. 2024 Jul 4;12(7):490. doi: 10.3390/toxics12070490 (PMC11281202; doi:10.3390/toxics12070490)
Supplement: Supplementary file 1 [file toxics-12-00490-s001.zip › toxics-3074115-supplementary.pdf]

**Biomonitoring Study of Toxic Metal(loid)s: Levels in Lung Adenocarcinoma Patients**

Nataša Milošević[1], Maja Milanović[1*], Danica Sazdanić Velikić[2], Jan Sudji[3], Jelena Jovičić-Bata[1], Milorad Španović[3], Mirjana Ševo[4,5], Mirka Lukić Šarkanović[6], Ljilja Torović[1], Sanja Bijelović[7], Nataša Milić[1]

[1]University of Novi Sad, Faculty of Medicine, Department of Pharmacy, Novi Sad, Serbia

[2]University of Novi Sad, Faculty of Medicine, Institute for pulmonary diseases of Vojvodina, Clinic for pulmonary oncology, Sremska Kamenica, Serbia

[3]University of Novi Sad, Faculty of Medicine, Institute of Occupational Health Novi Sad, Novi Sad, Serbia

[4]University of Novi Sad, Faculty of Medicine, Novi Sad, Serbia

[5]IMC Banja Luka-Center of Radiotherapy, part of Affidea group, Banja Luka, Bosnia and Herzegovina

[6]University of Novi Sad, Faculty of Medicine, University Clinical Center of Vojvodina, Clinic for anesthesiology, intensive therapy and pain therapy, Novi Sad, Serbia

[7]University of Novi Sad, Faculty of Medicine, Institute of Public Health of Vojvodina, Novi Sad, Serbia

*Corresponding author at: Department of Pharmacy, Faculty of Medicine, University of Novi Sad, Hajduk Veljkova 3, 21000 Novi Sad, Serbia.

E-mail address: maja.milanovic@mf.uns.ac.rs

**Table S1**. Sample preparation and ICP-MS instrument operating conditions.

| **ICP-MS** | | |
|---|---|---|
| *Sample preparation* | | |
| | A urine aliquot (1 mL) was placed in quartz inserts together with the 5 mL of nitric acid, while a mixture of 1 mL of hydrogen peroxide with 5 mL of deionized water was placed in teflon cuvette. The quartz inserts were carefully transferred into the teflon cuvettes ensuring that there was no physical contact with the samples. Afterwards, teflon cuvettes were closed and placed in a microwave oven and the following parameters were used for mineralization: temperature 200°C, applied power max. 1000 W and time 30 min. After the digestion and cooling, the mineralized samples were quantitatively transferred into graduated plastic tubes of 15 mL and diluted with deionized water to a total volume of 12 mL. A blank was prepared as urine using deionized water (1 ml aliquot). All glassware was rinsed with nitric acid before the use and after the mineral digestion. | |
| *Instrument operating conditions* | | |
| *Parameter* | Analysis mode | |
| | No Gas mode | He mode |
| RF- power (W) | 1550 | 1550 |
| Sample depth (mm) | 8 | 8 |
| Nebulizer pump speed (rps) | 0.1 | 0.1 |
| Plazma gas flow (L/min) | 15.0 | 15.0 |
| Carrier gas flow (L/min) | 1.2 | 1.2 |
| Dilution gas flow (L/min) | 1.0 | 1.0 |
| Spray chamber | Water cooled double pass | |
| Lens voltage | 4.5 | 4.5 |
| Mass resolution | 0.8 | 0.8 |
| Integration time points/ms | 3 | 3 |
| Points per peak | 3 | 3 |
| Replicates | 3 | 3 |

*Notes:* Prior to the analysis, the ICP-MS system was equilibrated for 30 min and then checked in terms of sensitivity, stability and performance, using the diluted tune solution (1 µg/L of each element), whereas auto-tune and calibration mass tests were performed when it was necessary for optimisation of the instrument. Internal standard was added at a constant rate and concentration to all calibration standards and unknown samples.

*Chemicals:* Nitric acid (Fisher Chemicals, UK); $H_2O_2$ (Fisher Chemicals, UK); Tuning solution: cerium (Ce), cobalt (Co), lithium (Li), magnesium (Mg), thallium (Tl), and ittrium (Y) (10 µg/mL each, in 2% v/v $HNO_3$) (Agilent Technologies, Waldbronn, Germany); Internal standard: the mixture of bismut (Bi), germanium (Ge), indium (In), lithium (Li), lutetium (Lu), rhodium (Rh), scandium (Sc), and terbium (Tb), (10 µg/mL each, in 10% v/v $HNO_3$) (Agilent Technologies, Waldbronn, Germany).

*Instruments:* Microwave oven (Start D Microwave Digestion System, Milestone, Brondby, Denmark), GenPure Water Purification System (Thermo Fisher Scientific, Langenselbold, Germany).

**Table S2**. ICP-MS monitored and reported isotope(s), analysis mode and corresponding internal standard for each element

| Element[a] | Symbol | Monitored isotopes | Reported isotope | Internal standard (isotope) | Analysis mode |
|---|---|---|---|---|---|
| Chromium | Cr | 50, 52 | 52 | Sc (45) | He |
| Manganese | Mn | 55 | 55 | Sc (45) | He |
| Nickel | Ni | 60, 61 | 60 | Sc (45) | He |
| Arsenic | As | 75 | 75 | Ge (74) | He |
| Molybdenum | Mo | 95 | 95 | Ge (74) | He |
| Cadmium | Cd | 111, 114 | 111 | In (115) | He |
| Mercury | Hg | 200, 201, 202 | average | Bi (209) | No gas |
| Lead | Pb | 206, 207, 208 | average | Bi (209) | No gas |

[a] The mix standard solutions were prepared by mixing and diluting of individual stock standard solutions of elements (1 g/L) with ultrapure water and nitric acid. Standard solutions of Cr, Ni, Mo and Cd were purchased from CPAchem (Stara Zagora, Bulgaria), Mn, As and Pb from AccuStandard Inc. (New Haven, CT, USA) and Hg from Carlo Erba (Milan, Italy).

**Table S3**. The anthropometric characteristics, blood pressure and laboratory findings of the enrolled patients presented as mean values with standard deviation, as well as smoking habits, belonging into family of smokers, exposure to second-hand smoking and presence of amalgam dental fillings given as percentage

| Parameter | **Total (n=63)** | **Male (n=36)** | **Female (n=27)** |
|---|---|---|---|
| Age (years) | 64.97±8.987 | 66.89±8.585 | 62.41±9.027 |
| BMI (kg/m$^2$) | 24.00±4.638 | 24.046±4.153 | 23.952±5.284 |
| Waist circumference (cm) | 88.38±13.70 | 90.88±13.45 | 85.33±13.630 |
| Hips circumference (cm) | 95.90±10.62 | 94.55±11.0524 | 97.56±10.02 |
| Waist-to-height ratio (cm/cm) | 0.510±0.126 | 0.496±0.146 | 0.53±0.09 |
| Waist-to-hips ratio (cm/cm) | 0.921±0.094 | 0.962±0.087 | 0.872±0.078 |
| Systolic blood pressure (mmHg) | 124.90±17.515 | 127.68±17.178 | 121.27±17.615 |
| Diastolic blood pressure (mmHg) | 74.80±8.628 | 75.15±8.522 | 74.35±8.908 |
| Red blood cells (×10$^{12}$/L) | 4.568±0.781 | 4.725±0.884 | 4.359±0.567 |
| White blood cells (×10$^9$/L) | 9.852±3.650 | 9.430±3.954 | 10.415±3.186 |
| Neutrophils (×10$^9$/L) | 7.299±3.170 | 7.006±3.444 | 7.690±2.779 |
| Neutrophils (%) | 72.44±8.070 | 72.08±8.292 | 72.93±7.893 |
| Lymphocytes (×10$^9$/L) | 1.65±0.732 | 1.440±0.524 | 1.929±0.875 |
| Lymphocytes (%) | 17.51±6.92 | 16.31±6.940 | 19.11±6.687 |
| Monocytes (×10$^9$/L) | 0.733±0.275 | 0.801±0.290 | 0.642±0.227 |
| Monocytes (%) | 7.714±2.511 | 8.694±2.265 | 6.407±2.241 |
| Eosinophils (×10$^9$/L) | 0.180±0.334 | 0.233±0.429 | 0.110±0.903 |
| Eosinophils (%) | 1.739±2.661 | 2.213±3.380 | 1.107±0.887 |
| Basophils (×10$^9$/L) | 0.058±0.032 | 0.059±0.032 | 0.057±0.032 |
| Basophils (%) | 0.596±0.306 | 0.621±0.288 | 0.563±0.331 |
| Hgb (g/L) | 129.54±18.352 | 131.89±18.131 | 126.41±18.515 |
| Hct (L/L) | 0.389±0.050 | 0.398±0.050 | 0.376±0.053 |
| MCV (fL) | 86.44±6.490 | 86.44±6.971 | 68.44±5.918 |
| MCH (pg) | 28.837±2.655 | 28.694±2.916 | 29.026±2.301 |
| MCHC (g/L) | 333.38±8.940 | 331.58±9.889 | 335.78±6.963 |
| Platelets (×10$^9$/L) | 337.30±108.545 | 314.39±9.478 | 367.85±114.361 |
| ALT (μkat/L) | 0.323±0.228 | 0.2894±0.126 | 0.3677±0.315 |
| AST (μkat/L) | 0.340±0.169 | 0.3197±0.114 | 0.3665±0.226 |
| GGT (μkat/L) | 0.682±0.560 | 0.6997±0.521 | 0.6576±0.619 |
| LDH (μkat/L) | 7.835±4.15 | 7.8397±4.603 | 7.830±3.557 |
| ALP (μkat/L) | 1.63±0.742 | 1.618±0.764 | 1.646±0.727 |
| CRP (mg/L) | 40.424±56.823 | 48.761±62.725 | 29.842±47.406 |
| Former smokers | 46.03% (29/63) | 52.78% (19/36) | 40.74% (11/27) |
| Current smokers | 46.03% (29/63) | 38.89% (14/36) | 51.85% (14/27) |
| Never smokers | 7.94 (5/63) | 8.33% (3/36) | 7.41% (2/27) |
| Family of smokers | 66.67% (42/63) | 66.67% (24/36) | 66.67% (18/27) |
| Family of never smokers | 33.33% (21/63) | 33.33% (12/36) | 33.33% (9/27) |
| Everyday second-hand smoking | 53.97% (34/63) | 52.78% (19/36) | 55.56% (15/27) |
| Rare/none second-hand smoking | 46.03% (29/63) | 47.22% (17/36) | 44.44% (12/27) |
| Amalgam dental fillings | 44.44% (28/63) | 38.89% (14/36) | 51.85% (14/27) |

BMI – body mass index, Hgb – hemoglobin, Hct -hematocrit, MCV – mean corpuscular volume, MCH – mean corpuscular Hgb, MCHC – mean corpuscular Hgb concentration, ALT - alanine aminotransferase, AST - aspartate aminotransferase, GGT - γ-glutamiltransferase, LDH - lactate dehydrogenase, ALP - alkaline phosphatase and CRP - C-reactive protein

**Table S4**. The difference in anthropometric characteristics, blood pressure and laboratory findings of the patients with urinary chromium, manganese, nickel and arsenic concentration above and below LOQ

| | Chromium | | Manganese | | Nickel | | Arsenic | |
|---|---|---|---|---|---|---|---|---|
| | **≥LOQ (n=26)** | **<LOQ (n=37)** | **≥LOQ (n=20)** | **<LOQ (n=43)** | **≥LOQ (n=32)** | **<LOQ (n=31)** | **≥LOQ (n=58)** | **<LOQ (n=5)** |
| Age (years) | 64.04±9.966 | 65.62±8.311 | 66.20±9.294 | 64.40±8.894 | 65.03±9.597 | 64.90±8.471 | 64.90±9.208 | 65.80±6.535 |
| BMI (kg/m$^2$) | 23.961±5.592 | 24.037±3.892 | 23.769±5.365 | 24.118±4.316 | 23.897±4.775 | 24.120±4.566 | 24.099±4.636 | 22.929±5.057 |
| Waist circumference (cm) | 87.92±15.300 | 88.71±12.657 | 88.25±14.209 | 88.45±13.623 | 87.25±14.602 | 89.68±12.731 | 89.04±13.613 | 79.25±13.226 |
| Hips circumference (cm) | 96.28±10.945 | 95.63±10.533 | 96.65±10.999 | 95.53±10.547 | 94.47±11.359 | 97.54±9.647 | 96.00±10.425 | 94.50±14.911 |
| WtHR(cm/cm) | 0.508±0.144 | 0.512±0.113 | 0.528±0.095 | 0.502±0.138 | 0.525±0.925 | 0.495±0.154 | **0.522±0.109** | **0.382±0.228*** |
| WtHipR ratio(cm/cm) | 0.9108±0.096 | 0.929±0.093 | 0.9112±0.095 | 0.926±0.0946 | 0.923±0.095 | 0.919±0.095 | 0.927±0.093 | 0.840±0.802 |
| Systolic BP (mmHg) | 124.71±20.395 | 125.03±15.612 | 127.50±22.897 | 123.79±14.827 | 125.09±17.842 | 124.68±17.457 | 125.53±17.904 | 118.00±11.511 |
| Diastolic BP (mmHg) | 76.00±7.299 | 74.03±9.398 | 74.18±8.784 | 75.05±8.659 | 75.39±7.894 | 74.14±9.478 | 74.67±8.707 | 76.20±8.497 |
| Red blood cells (×10$^{12}$/L) | 4.384±0.557 | 4.698±0.890 | 4.414±0.56 | 4.640±0.861 | 4.643±0.980 | 4.491±0.504 | 4.569±0.809 | 4.572±0.341 |
| White blood cells (×10$^9$/L) | 9.861±3.200 | 9.845±3.979 | 9.770±3.169 | 9.890±3.889 | 9.453±3.141 | 10.264±4.122 | 9.863±3.730 | 9.720±2.877 |
| Neutrophils (×10$^9$/L) | 7.308±2.615 | 7.293±3.544 | 7.269±2.789 | 7.314±3.364 | 7.045±2.526 | 7.56±3.747 | 7.170±2.580 | 7.310±3.235 |
| Neutrophils (%) | 73.73±6.972 | 71.54±8.379 | 73.50±7.612 | 71.95±8.315 | 73.09±7.279 | 71.77±8.883 | 72.45±8.300 | 72.40±5.273 |
| Lymphocytes (×10$^9$/L) | 1.655±0.932 | 1.646±0.565 | 1.628±0.719 | 1.660±0.746 | 1.654±0.860 | 1.644±0.584 | 1.645±0.759 | 1.696±0.311 |
| Lymphocytes (%) | 16.77±6.671 | 18.03±7.136 | 17.15±6.877 | 17.67±7.016 | 17.53±0.325 | 17.48±7.028 | 17.45±7.131 | 18.20±4.147 |
| Monocytes (×10$^9$/L) | 0.7154±0.210 | 0.745±0.315 | 0.7275±0.206 | 0.735±0.304 | 0.684±0.209 | 0.783±0.325 | 0.7305±0.284 | 0.758±0.143 |
| Monocytes (%) | 7.539±2.231 | 7.838±2.713 | 7.75±1.97 | 7.770±2.747 | 7.406±2.284 | 8.02±2.726 | 7.672±2.557 | 8.200±2.049 |
| Eosinophils (×10$^9$/L) | 0.1358±0.144 | 0.211±0.418 | **0.095±0.085** | **0.219±0.035#** | 0.139±0.133 | 0.222±0.456 | 0.190±0.346 | 0.066±0.063 |
| Eosinophils (%) | 1.389±1.495 | 1.986±3.240 | **1.00±0.837** | **2.083±3.124*** | 1.509±1.492 | 1.977±3.496 | 1.827±2.753 | 0.72±0.589 |
| Basophils (×10$^9$/L) | 0.0515±0.178 | 0.0624±0.039 | 0.054±0.026 | 0.0598±0.035 | 0.053±0.243 | 0.063±0.040 | 0.0597±0.033 | 0.038±0.011 |
| Basophils (%) | 0.5325±0.239 | 0.6405±0.341 | 0.562±0.305 | 0.612±0.309 | 0.551±0.243 | 0.642±0.358 | 0.611±0.309 | 0.420±0.217 |
| Hgb (g/L) | 127.5±18.945 | 130.97±18.047 | 124.95±17.86 | 131.67±18.39 | 129.50±20.181 | 129.58±16.589 | 129.22±18.721 | 133.20±14.412 |
| Hct (L/L) | 0.380±0.0545 | 0.395±0.05 | 0.374±0.051 | 0.395±0.052 | 0.388±0.0594 | 0.389±0.044 | 0.388±0.054 | 0.392±0.035 |
| MCV (fL) | 86.77±6.930 | 86.22±6.25 | 84.90±6.68 | 87.16±6.351 | 86.09±6.836 | 86.81±6.204 | 86.47±6.492 | 86.20±7.225 |
| MCH (pg) | 29.165±2.767 | 28.605±2.586 | 28.385±22.706 | 29.047±2.636 | 28.734±2.660 | 28.942±2.69 | 28.803±2.629 | 29.220±3.254 |
| MCHC (g/L) | 335.77±7.426 | 331.70±9.609 | 334.15±7.45 | 333.02±9.615 | 333.69±6.898 | 333.06±10.761 | 332.88±8.633 | 339.20±11.432 |
| Platelets (×10$^9$/L) | 323.19±116.03 | 347.22±103.424 | 350.50±126.16 | 331.16±100.35 | 326.13±110.654 | 348.84±106.894 | 337.69±109.108 | 332.80±113.819 |
| ALT (μkat/L) | **0.401±0.327** | **0.265±0.071*** | 0.385±0.332 | 0.292±0.151 | 0.340±0.235 | 0.304±0.223 | 0.309±0.185 | 0.476±0.525 |
| AST (μkat/L) | 0.377±0.218 | 0.312±0.116 | 0.345±0.119 | 0.337±0.19 | 0.364±0.157 | 0.312±0.132 | 0.339±0.170 | 0.344±0.183 |
| GGT (μkat/L) | 0.655±0.576 | 0.7014±0.555 | 0.708±0.441 | 0.670±0.612 | 0.675±0.538 | 0.691±0.593 | 0.706±0.576 | 0.422±0.221 |
| LDH (μkat/L) | 7.725±3.23 | 7.913±4.739 | 8.019±4.027 | 7.753±4.252 | 8.000±3.514 | 7.659±4.800 | 7.939±4.277 | 6.437±1.117 |
| ALP (μkat/L) | 1.607±0.781 | 1.647±0.727 | 1.585±0.452 | 1.652±0.848 | 1.544±0.718 | 1.730±0.770 | 1.640±0.772 | 1.532±0.268 |
| CRP (mg/L) | 45.24±59.734 | 36.882±55.227 | 53.147±63.884 | 34.380±52.937 | 38.141±55.905 | 42.360±58.571 | 40.222±57.371 | 42.600±56.611 |

*Statistically significant difference $p<0.05$ #p=0.055

**Table S5**. The difference in anthropometric characteristics, blood pressure and laboratory findings of the patients with urinary molybdenum, cadmium, mercury and lead concentration above and below LOQ

| | **Molybdenum** | | **Cadmium** | | **Mercury** | | **Lead** | |
|---|---|---|---|---|---|---|---|---|
| | **≥LOQ (n=57)** | **<LOQ (n=6)** | **≥LOQ (n=39)** | **<LOQ (n=24)** | **≥LOQ (n=40)** | **<LOQ (n=23)** | **≥LOQ (n=6)** | **<LOQ (n=57)** |
| Age (years) | 64.44±9.028 | 70.00±7.43 | 65.03±8.701 | 64.88±9.625 | 64.40±10.038 | 65.96±6.885 | 59.83±12.671 | 65.51±8.483 |
| BMI (kg/m$^2$) | 24.316±4.740 | 21.106±2.030 | 24.114±4.901 | 23.820±4.255 | 23.992±4.456 | 24.027±5.036 | 25.489±7.637 | 23.846±4.277 |
| Waist circumference (cm) | 89.17±13.715 | 81.33±2.437 | 87.30±13.766 | 90.13±13.716 | 88.18±12.475 | 88.73±15.905 | 86.17±18.324 | 88.63±13.291 |
| Hips circumference (cm) | 96.24±10.849 | 92.83±8.400 | 95.73±11.827 | 96.17±8.569 | 95.82±11.213 | 96.05±9.761 | 98.00±13.115 | 95.67±10.429 |
| WtHR(cm/cm) | 0.514±0.131 | 0.473±0.044 | 0.494±0.17 | 0.537±0.074 | 0.513±0.114 | 0.506±0.1465 | 0.536±0.143 | 0.508±0.125 |
| WtHipR ratio(cm/cm) | 0.926±0.095 | 0.874±0.081 | 0.912±0.0867 | 0.936±0.105 | 0.922±0.086 | 0.920±0.109 | 0.874±0.084 | 0.926±0.094 |
| Systolic BP (mmHg) | 124.80±17.306 | 125.83±21.075 | 123.79±18.920 | 126.82±15.004 | 124.84±18.671 | 125.00±15.736 | 127.50±30.943 | 124.61±15.821 |
| Diastolic BP (mmHg) | 75.06±8.020 | 72.50±13.693 | 74.03±8.635 | 76.19±8.646 | 74.81±8.569 | 74.77±8.928 | 71.67±6.831 | 75.15±8.791 |
| Red blood cells (×10$^{12}$/L) | 4.581±0.800 | 4.447±0.603 | 4.527±0.820 | 4.634±0.724 | 4.613±0.897 | 4.490±0.530 | 4.455±0.347 | 4.580±0.814 |
| White blood cells (×10$^9$/L) | 9.973±3.737 | 8.700±2.650 | 9.245±2.856 | 10.838±4.561 | 9.540±3.115 | 10.396±4.457 | 4.455±0.347 | 4.580±0.814 |
| Neutrophils (×10$^9$/L) | 7.362±3.250 | 6.707±2.427 | 6.857±2.472 | 8.017±4.016 | 7.133±2.904 | 7.588±3.638 | 10.583±2.960 | 9.775±3.729 |
| Neutrophils (%) | 72.07±8.170 | 76.00±6.573 | 72.44±7.601 | 72.46±8.949 | 72.45±8.978 | 72.43±6.373 | 71.33±9.416 | 72.56±8.002 |
| Lymphocytes (×10$^9$/L) | 1.689±0.747 | 1.280±0.450 | 1.567±0.647 | 1.784±0.850 | 1.557±0.542 | 1.81±0.972 | 2.090±0.864 | 1.603±0.709 |
| Lymphocytes (%) | 17.75±7.001 | 15.17±6.145 | 17.31±6.818 | 17.83±7.221 | 17.27±6.928 | 17.91±7.045 | 20.67±7.659 | 17.18±6.827 |
| Monocytes (×10$^9$/L) | 0.746±0.282 | 0.603±0.157 | 0.727±0.274 | 0.743±0.281 | 0.720±0.231 | 0.755±0.342 | 0.600±0.091 | 0.747±0.284 |
| Monocytes (%) | 7.772±2.584 | 7.167±1.722 | 7.949±2.70 | 7.333±2.160 | 7.850±2.517 | 7.478±2.538 | 5.833±1.472 | 7.912±2.523 |
| Eosinophils (×10$^9$/L) | 0.191±0.348 | 0.070±0.060 | 0.146±0.171 | 0.235±0.496 | 0.1745±0.279 | 0.189±0.419 | 0.162±0.074 | 0.182±0.350 |
| Eosinophils (%) | 1.822±2.775 | 0.950±0.855 | 1.620±2.019 | 1.933±3.506 | 1.890±2.886 | 1.478±2.254 | 1.700±1.070 | 1.743±2.782 |
| Basophils (×10$^9$/L) | 0.059±0.033 | 0.050±0.0167 | 0.0531±0.027 | 0.0658±0.038 | 0.0565±0.317 | 0.060±0.042 | 0.053±0.008 | 0.058±0.0334 |
| Basophils (%) | 0.597±0.319 | 0.583±0.147 | 0.587±0.312 | 0.610±0.302 | 0.6135±0.302 | 0.565±0.317 | 0.5167±0.248 | 0.6042±0.312 |
| Hgb (g/L) | 128.75±18.577 | 137.00±15.401 | 128.10±14.876 | 131.88±23.088 | 128.48±18.246 | 131.39±18.797 | 123.50±11.845 | 130.18±18.869 |
| Hct (L/L) | 0.387±0.053 | 0.402±0.043 | 0.384±0.041 | 0.397±0.066 | 0.387±0.053 | 0.391±0.052 | 0.368±0.029 | 0.391±0.053 |
| MCV (fL) | 85.98±6.602 | 90.83±2.927 | 86.97±6.347 | 85.58±6.763 | 86.05±6.702 | 87.13±6.189 | 83.17±8.353 | 86.79±6.256 |
| MCH (pg) | **28.614±2.676*** | **30.950±1.141*** | 29.044±2.586 | 28.500±2.787 | 28.580±2.721 | 2.283±2.532 | 27.867±3.4174 | 28.939±2.579 |
| MCHC (g/L) | **332.54±8.944*** | **341.33±3.327*** | 333.92±8.424 | 332.50±9.842 | 332.10±8.521 | 335.61±9.400 | 335.17±7.910 | 333.19±9.084 |
| Platelets (×10$^9$/L) | 345.60±108.22 | 258.50±81.777 | 328.59±120.418 | 351.46±86.403 | 351.80±117.29 | 312.09±88.149 | 370.67±170.061 | 333.79±101.639 |
| ALT (µkat/L) | 0.3313±0.237 | 0.2450±0.082 | 0.334±0.265 | 0.304±0.143 | 0.3346±0.269 | 0.3018±0.131 | 0.373±0.412 | 0.317±0.204 |
| AST (µkat/L) | 0.342±0.175 | 0.298±0.097 | 0.340±0.179 | 0.3338±0.154 | 0.3449±0.1854 | 0.3305±0.139 | 0.307±0.084 | 0.343±0.176 |
| GGT (µkat/L) | 0.709±0.581 | 0.442±0.190 | 0.648±0.510 | 0.746±0.651 | 0.7116±0.524 | 0.6314±0.626 | 0.397±0.205 | 0.714±0.578 |
| LDH (µkat/L) | 7.895±4.345 | 7.323±1.851 | 8.077±3.148 | 7.440±5.475 | 7.663±3.332 | 8.139±5.381 | 8.222±2.999 | 7.781±4.284 |
| ALP (µkat/L) | 1.660±0.774 | 1.367±0.251 | 1.679±0.696 | 1.547±0.827 | 1.628±0.702 | 1.635±0.824 | 1.740±0.354 | 1.618±0.775 |
| CRP (mg/L) | 40.302±54.367 | 41.500±81.958 | 42.800±58.126 | 36.124±55.533 | 40.135±50.448 | 40.909±67.493 | 33.667±37.628 | 41.189±58.820 |

*Statistically significant difference $p<0.05$

**Table S6**. The statistical parameters of the correlations between the urinary metal(loid)s concentration in μg/L and anthropometric characteristics, blood pressure and laboratory findings

| | **Cr (μg/L)** | | **Mn (μg/L)** | | **Ni (μg/L)** | | **As (μg/L)** | | **Mo (μg/L)** | | **Cd (μg/L)** | | **Hg (μg/L)** | | **Pb (μg/L)** | |
|---|---|---|---|---|---|---|---|---|---|---|---|---|---|---|---|---|
| | **Adj. $r^2$** | **p** | **Adj. $r^2$** | **p** | **Adj. $r^2$** | **p** | **Adj. $r^2$** | **p** | **Adj. $r^2$** | **p** | **Adj. $r^2$** | **p** | **Adj. $r^2$** | **p** | **Adj. $r^2$** | **p** |
| Age (years) | 0.064 | 0.114 | 0.008 | 0.297 | -0.031 | 0.804 | 0.002 | 0.294 | -0.007 | 0.439 | -0.014 | 0.503 | -0.022 | 0.674 | 0.273 | 0.165 |
| BMI (kg/m$^2$) | -0.038 | 0.768 | -0.026 | 0.482 | -0.030 | 0.772 | -0.009 | 0.474 | -0.018 | 0.935 | -0.023 | 0.711 | 0.017 | 0.208 | -0.116 | 0.527 |
| Waist circumfer. (cm) | -0.011 | 0.402 | -0.009 | 0.373 | -0.021 | 0.558 | -0.018 | 0.964 | -0.017 | 0.716 | -0.026 | 0.761 | -0.017 | 0.537 | -0.248 | 0.938 |
| Hips circumfer. (cm) | -0.034 | 0.658 | -0.027 | 0.488 | 0.005 | 0.294 | -0.015 | 0.661 | -0.004 | 0.386 | 0.070 | 0.062 | -0.002 | 0.346 | -0.248 | 0.940 |
| WtHR(cm/cm) | -0.029 | 0.586 | -0.049 | 0.751 | -0.019 | 0.523 | -0.018 | 0.940 | -0.018 | 0.873 | -0.015 | 0.507 | -0.026 | 0.862 | -0.183 | 0.659 |
| WtHipR ratio(cm/cm) | **0.129** | **0.044** | -0.043 | 0.654 | -0.030 | 0.762 | -0.016 | 0.739 | -0.013 | 0.573 | **0.136** | **0.014** | -0.026 | 0.830 | -0.227 | 0.799 |
| Systolic BP (mmHg) | -0.047 | 0.157 | -0.048 | 0.643 | -0.028 | 0.698 | -0.017 | 0.762 | -0.003 | 0.372 | -0.023 | 0.685 | -0.002 | 0.340 | -0.166 | 0.619 |
| Diastolic BP (mmHg) | -0.048 | 0.982 | -0.034 | 0.504 | 0.008 | 0.277 | 0.050 | 0.056 | -0.009 | 0.464 | 0.003 | 0.295 | 0.015 | 0.223 | -0.148 | 0.584 |
| RBC ($\times10^{12}$/L) | 0.020 | 0.229 | -0.047 | 0.697 | -0.032 | 0.842 | -0.010 | 0.523 | -0.010 | 0.220 | **0.350** | **<0.001** | -0.006 | 0.389 | -0.206 | 0.723 |
| WBC ($\times10^9$/L) | -0.039 | 0.804 | -0.050 | 0.175 | -0.030 | 0.746 | -0.007 | 0.445 | <0.001 | 0.317 | 0.001 | 0.316 | **0.157** | **0.007** | -0.241 | 0.872 |
| Neutrophils ($\times10^9$/L) | -0.040 | 0.854 | 0.061 | 0.153 | -0.031 | 0.785 | -0.004 | 0.377 | -0.007 | 0.439 | -0.023 | 0.706 | **0.102** | **0.025** | -0.091 | 0.487 |
| Neutrophils (%) | -0.041 | 0.932 | -0.045 | 0.679 | -0.031 | 0.789 | 0.022 | 0.138 | -0.015 | 0.657 | 0.019 | 0.196 | -0.025 | 0.834 | 0.372 | 0.118 |
| Lymphocytes ($\times10^9$/L) | -0.034 | 0.675 | -0.052 | 0.811 | -0.022 | 0.571 | -0.016 | 0.771 | -0.010 | 0.510 | 0.001 | 0.316 | 0.021 | 0.183 | 0.416 | 0.10 |
| Lymphocytes (%) | -0.037 | 0.757 | -0.053 | 0.849 | -0.032 | 0.860 | -0.005 | 0.403 | -0.006 | 0.421 | 0.013 | 0.226 | -0.010 | 0.444 | 0.391 | 0.11 |
| Monocytes ($\times10^9$/L) | -0.002 | 0.342 | 0.030 | 0.225 | -0.033 | 0.955 | -0.012 | 0.577 | -0.014 | 0.651 | <0.001 | 0.325 | 0.083 | 0.040 | 0.083 | 0.294 |
| Monocytes (%) | -0.003 | 0.345 | -0.055 | 0.974 | 0.002 | 0.313 | 0.011 | 0.206 | -0.015 | 0.691 | 0.002 | 0.305 | -0.026 | 0.983 | -0.123 | 0.538 |
| Eosinophils ($\times10^9$/L) | -0.027 | 0.569 | -0.001 | 0.331 | 0.008 | 0.273 | -0.010 | 0.520 | -0.016 | 0.762 | -0.027 | 0.954 | 0.025 | 0.167 | 0.501 | 0.070 |
| Eosinophils (%) | -0.022 | 0.505 | 0.063 | 0.149 | -0.008 | 0.394 | -0.001 | 0.336 | -0.018 | 0.978 | -0.026 | 0.874 | 0.006 | 0.276 | 0.119 | 0.266 |
| Basophils ($\times10^9$/L) | -0.019 | 0.475 | -0.039 | 0.603 | **0.108** | **0.037** | -0.014 | 0.626 | -0.011 | 0.524 | -0.026 | 0.907 | 0.027 | 0.156 | -0.160 | 0.607 |
| Basophils (%) | -0.040 | 0.834 | 0.043 | 0.189 | **0.232** | **0.003** | 0.009 | 0.223 | 0.024 | 0.131 | -0.023 | 0.717 | -0.023 | 0.736 | -0.226 | 0.792 |
| Hgb (g/L) | 0.060 | 0.120 | -0.040 | 0.608 | -0.030 | 0.760 | -0.012 | 0.561 | 0.014 | 0.186 | -0.026 | 0.870 | -0.013 | 0.482 | -0.250 | 0.985 |
| Hct (L/L) | 0.077 | 0.092 | -0.036 | 0.571 | -0.030 | 0.753 | -0.011 | 0.555 | 0.015 | 0.178 | -0.024 | 0.755 | -0.004 | 0.369 | -0.245 | 0.902 |
| MCV (fL) | 0.010 | 0.275 | -0.049 | 0.748 | -0.031 | 0.804 | -0.010 | 0.519 | 0.011 | 0.210 | -0.027 | 0.978 | 0.003 | 0.296 | -0.228 | 0.801 |
| MCH (pg) | -0.009 | 0.387 | -0.053 | 0.838 | -0.033 | 0.914 | -0.012 | 0.551 | <0.001 | 0.317 | -0.016 | 0.531 | 0.006 | 0.273 | -0.225 | 0.788 |
| MCHC (g/L) | -0.034 | 0.800 | -0.055 | 0.931 | -0.033 | 0.962 | -0.018 | 0.962 | -0.017 | 0.821 | 0.070 | 0.057 | -0.010 | 0.439 | -0.190 | 0.677 |
| Platelets ($\times10^9$/L) | -0.027 | 0.572 | -0.030 | 0.516 | 0.006 | 0.284 | -0.017 | 0.870 | -0.017 | 0.794 | -0.002 | 0.346 | -0.009 | 0.425 | -0.110 | 0.516 |
| ALT (μkat/L) | -0.031 | 0.628 | 0.010 | 0.289 | -0.028 | 0.699 | -0.016 | 0.732 | -0.006 | 0.408 | -0.004 | 0.367 | -0.021 | 0.648 | -0.143 | 0.574 |
| AST (μkat/L) | -0.016 | 0.447 | 0.012 | 0.282 | -0.033 | 0.968 | 0.002 | 0.292 | -0.005 | 0.391 | -0.006 | 0.389 | **0.119** | **0.018** | -0.061 | 0.446 |
| GGT (μkat/L) | -0.034 | 0.650 | -0.039 | 0.581 | -0.032 | 0.876 | 0.016 | 0.176 | -0.011 | 0.514 | -0.027 | 0.918 | **0.161** | **0.073** | -0.067 | 0.454 |
| LDH (μkat/L) | -0.045 | 0.986 | -0.033 | 0.509 | -0.035 | 0.931 | -0.003 | 0.362 | -0.019 | 0.868 | -0.002 | 0.342 | -0.014 | 0.476 | -0.249 | 0.982 |
| ALP (μkat/L) | 0.033 | 0.189 | -0.051 | 0.731 | -0.032 | 0.860 | 0.003 | 0.280 | -0.019 | 0.874 | -0.027 | 0.919 | 0.026 | 0.166 | -0.179 | 0.648 |
| CRP (mg/L) | -0.033 | 0.637 | -0.041 | 0.602 | 0.010 | 0.265 | -0.002 | 0.355 | 0.004 | 0.277 | -0.025 | 0.751 | -0.028 | 0.863 | -0.194 | 0.687 |

**Table S7**. The statistical parameters of the correlations between the urinary metal(oid)s concentration in µg/gCre and anthropometric characteristics, blood pressure and laboratory findings

| | **Cr (µg/gCre)** | | **Mn (µg/ gCre)** | | **Ni (µg/gCre)** | | **As (µg/gCre)** | | **Mo (µg/gCre)** | | **Cd (µg/gCre)** | | **Hg (µg/gCre)** | | **Pb (µg/gCre)** | |
|---|---|---|---|---|---|---|---|---|---|---|---|---|---|---|---|---|
| | **Adj. $r^2$** | **p** | **Adj. $r^2$** | **p** | **Adj. $r^2$** | **p** | **Adj. $r^2$** | **p** | **Adj. $r^2$** | **p** | **Adj. $r^2$** | **p** | **Adj. $r^2$** | **p** | **Adj. $r^2$** | **p** |
| Age (years) | -0.028 | 0.579 | -0.048 | 0.727 | -0.018 | 0.507 | -0.002 | 0.343 | -0.012 | 0.572 | -0.001 | 0.336 | -0.023 | 0.710 | 0.412 | 0.101 |
| BMI (kg/m²) | -0.026 | 0.550 | -0.029 | 0.506 | -0.013 | 0.445 | -0.018 | 0.926 | -0.003 | 0.374 | -0.026 | 0.833 | 0.021 | 0.188 | 0.083 | 0.295 |
| Waist circumfer. (cm) | -0.040 | 0.793 | -0.050 | 0.766 | -0.033 | 0.973 | -0.004 | 0.386 | -0.001 | 0.336 | -0.027 | 0.841 | 0.006 | 0.274 | -0.019 | 0.394 |
| Hips circumfer. (cm) | -0.043 | 0.935 | -0.053 | 0.850 | -0.015 | 0.466 | -0.018 | 0.869 | 0.011 | 0.211 | -0.020 | 0.601 | -0.022 | 0.663 | 0.141 | 0.248 |
| WtHR(cm/cm) | -0.041 | 0.904 | -0.004 | 0.350 | -0.033 | 0.973 | -0.018 | 0.901 | -0.017 | 0.804 | 0.015 | 0.215 | -0.023 | 0.697 | 0.010 | 0.284 |
| WtHipR ratio(cm/cm) | -0.033 | 0.630 | -0.046 | 0.693 | -0.010 | 0.411 | 0.024 | 0.128 | -0.019 | 0.982 | -0.021 | 0.612 | 0.015 | 0.218 | -0.234 | 0.829 |
| Systolic BP (mmHg) | -0.062 | 0.127 | -0.030 | 0.488 | -0.022 | 0.561 | -0.017 | 0.733 | -0.001 | 0.334 | -0.017 | 0.536 | -0.028 | 0.962 | -0.017 | 0.393 |
| Diastolic BP (mmHg) | -0.018 | 0.440 | 0.082 | 0.139 | -0.015 | 0.463 | 0.028 | 0.116 | -0.019 | 0.921 | -0.022 | 0.664 | 0.010 | 0.248 | 0.004 | 0.370 |
| RBC (×10¹²/L) | 0.015 | 0.251 | -0.047 | 0.714 | -0.014 | 0.455 | -0.016 | 0.745 | <0.001 | 0.319 | 0.014 | 0.225 | 0.003 | 0.301 | -0.238 | 0.855 |
| WBC (×10⁹/L) | -0.042 | 0.946 | -0.043 | 0.642 | -0.033 | 0.886 | -0.002 | 0.350 | 0.001 | 0.306 | -0.007 | 0.394 | -0.003 | 0.358 | -0.138 | 0.564 |
| Neutrophils (×10⁹/L) | -0.033 | 0.646 | -0.048 | 0.715 | -0.033 | 0.894 | -0.002 | 0.347 | -0.007 | 0.442 | -0.022 | 0.668 | -0.016 | 0.542 | -0.057 | 0.441 |
| Neutrophils (%) | 0.067 | 0.108 | -0.034 | 0.549 | -0.033 | 0.850 | -0.001 | 0.342 | -0.008 | 0.468 | -0.027 | 0.943 | -0.015 | 0.512 | -0.024 | 0.400 |
| Lymphocytes (×10⁹/L) | 0.007 | 0.289 | -0.048 | 0.714 | -0.031 | 0.805 | -0.017 | 0.868 | -0.015 | 0.669 | -0.027 | 0.892 | -0.012 | 0.463 | -0.224 | 0.787 |
| Lymphocytes (%) | 0.063 | 0.114 | -0.043 | 0.641 | -0.028 | 0.702 | 0.006 | 0.254 | -0.017 | 0.817 | -0.019 | 0.590 | -0.025 | 0.803 | -0.150 | 0.587 |
| Monocytes (×10⁹/L) | -0.028 | 0.575 | -0.004 | 0.350 | 0.027 | 0.184 | 0.001 | 0.304 | **0.057** | **0.041** | 0.009 | 0.256 | **0.110** | **0.021** | -0.010 | 0.499 |
| Monocytes (%) | -0.042 | 0.997 | -0.015 | 0.406 | 0.028 | 0.180 | -0.017 | 0.877 | 0.025 | 0.126 | 0.007 | 0.266 | **0.090** | **0.034** | 0.016 | 0.358 |
| Eosinophils (×10⁹/L) | -0.018 | 0.458 | -0.052 | 0.780 | <0.001 | 0.330 | -0.017 | 0.847 | -0.012 | 0.569 | -0.018 | 0.568 | -0.003 | 0.356 | 0.174 | 0.225 |
| Eosinophils (%) | -0.004 | 0.355 | -0.053 | 0.836 | -0.003 | 0.348 | -0.017 | 0.876 | -0.017 | 0.768 | -0.019 | 0.595 | -0.005 | 0.371 | 0.109 | 0.273 |
| Basophils (×10⁹/L) | 0.062 | 0.117 | -0.056 | 0.985 | 0.015 | 0.233 | -0.018 | 0.944 | -0.018 | 0.867 | -0.010 | 0.433 | -0.020 | 0.640 | -0.127 | 0.545 |
| Basophils (%) | -0.027 | 0.564 | -0.056 | 0.982 | 0.063 | 0.090 | -0.011 | 0.539 | -0.015 | 0.678 | -0.010 | 0.426 | -0.023 | 0.747 | -0.140 | 0.568 |
| Hgb (g/L) | -0.040 | 0.853 | -0.038 | 0.590 | -0.028 | 0.711 | 0.003 | 0.279 | 0.017 | 0.166 | -0.027 | 0.927 | -0.019 | 0.611 | -0.052 | 0.435 |
| Hct (L/L) | -0.039 | 0.815 | -0.039 | 0.602 | -0.026 | 0.656 | 0.007 | 0.40 | 0.019 | 0.154 | -0.024 | 0.732 | -0.012 | 0.470 | -0.086 | 0.481 |
| MCV (fL) | 0.064 | 0.113 | 0.091 | 0.106 | -0.032 | 0.831 | -0.011 | 0.529 | <0.001 | 0.333 | -0.015 | 0.508 | 0.006 | 0.271 | -0.143 | 0.574 |
| MCH (pg) | 0.052 | 0.137 | 0.068 | 0.140 | -0.033 | 0.952 | -0.014 | 0.654 | -0.006 | 0.414 | -0.025 | 0.779 | 0.016 | 0.211 | 0.123 | 0.538 |
| MCHC (g/L) | -0.035 | 0.689 | -0.046 | 0.682 | -0.023 | 0.585 | -0.015 | 0.681 | -0.018 | 0.851 | -0.008 | 0.403 | 0.002 | 0.306 | 0.074 | 0.464 |
| Platelets (×10⁹/L) | 0.059 | 0.123 | -0.112 | 0.080 | -0.033 | 0.905 | -0.012 | 0.577 | -0.011 | 0.537 | -0.009 | 0.417 | -0.006 | 0.383 | 0.262 | 0.171 |
| ALT (µkat/L) | -0.042 | 0.984 | -0.036 | 0.567 | -0.033 | 0.886 | -0.015 | 0.663 | -0.018 | 0.867 | -0.027 | 0.953 | **0.115** | **0.020** | -0.102 | 0.504 |
| AST (µkat/L) | -0.035 | 0.702 | 0.040 | 0.197 | 0.026 | 0.186 | -0.013 | 0.596 | -0.017 | 0.760 | -0.026 | 0.841 | **0.619** | **<0.001** | -0.015 | 0.391 |
| GGT (µkat/L) | -0.043 | 0.962 | 0.014 | 0.279 | 0.083 | 0.061 | 0.015 | 0.181 | -0.019 | 0.882 | -0.024 | 0.756 | **0.408** | **<0.001** | -0.056 | 0.440 |
| LDH (µkat/L) | -0.016 | 0.430 | -0.041 | 0.577 | -0.031 | 0.732 | -0.019 | 0.909 | -0.019 | 0.787 | -0.017 | 0.526 | -0.023 | 0.657 | 0.045 | 0.329 |
| ALP (µkat/L) | -0.039 | 0.762 | -0.041 | 0.599 | -0.018 | 0.508 | -0.001 | 0.337 | -0.009 | 0.473 | 0.021 | 0.189 | -0.024 | 0.721 | 0.367 | 0.120 |
| CRP (mg/L) | **0.139** | **0.037** | -0.057 | 0.883 | -0.028 | 0.644 | -0.013 | 0.570 | 0.027 | 0.124 | -0.014 | 0.479 | -0.026 | 0.769 | -0.125 | 0.541 |

**Table S8.** Estimate of the longest survival time ± standard error based on the Kaplan-Meier survival analysis upon the presence of each metal(oid) in the urine samples below or above LOQ values

| | **Cr** | **Mn** | **Ni** | **As** | **Mo** | **Cd** | **Hg** | **Pb** |
|---|---|---|---|---|---|---|---|---|
| <LOQ | 7.767±1.169 | 7.970±1.098 | 7.346±1.145 | 6.000±1.472 | 7.000±4.000 | 8.211±1.426 | 6.706±1.084 | 8.087±0.879 |
| ≥LOQ | 8.444±1.175 | 8.133±1.279 | 8.818±1.265 | 8.205±0.912 | 8.065±0.874 | 7.897±1.065 | 8.742±1.160 | 6.500±2.500 |
| p-value | 0.644 | 0.939 | 0.233 | 0.387 | 0.718 | 0.754 | 0.146 | 0.604 |
